# Proteomic profile of naturally released extracellular vesicles secreted from *Leptospira interrogans* serovar Pomona in response to temperature and osmotic stresses

**DOI:** 10.1038/s41598-023-45863-0

**Published:** 2023-10-30

**Authors:** Teerasit Techawiwattanaboon, Eakalak Phanchamnan, Nutta Iadsee, Jiradej Makjaroen, Trairak Pisitkun, Kanitha Patarakul

**Affiliations:** 1 Department of Microbiology, Faculty of Medicine, Chulalongkorn University, Bangkok, Thailand; 2 Chula Vaccine Research Center (Chula VRC), Center of Excellence in Vaccine Research and Development, Chulalongkorn University, Bangkok, Thailand; 3 Medical Microbiology, Interdisciplinary Program, Graduate School, Chulalongkorn University, Bangkok, Thailand; 4 Center of Excellence in Systems Biology, Faculty of Medicine, Chulalongkorn University, Bangkok, Thailand; 5 Department of Transfusion Medicine and Clinical Microbiology, Faculty of Allied Health Sciences, Chulalongkorn University, Bangkok, Thailand

**Keywords:** Microbiology, Bacteriology, Cellular microbiology

## Abstract

Bacterial extracellular vesicles (EVs) are generally formed by pinching off outer membrane leaflets while simultaneously releasing multiple active molecules into the external environment. In this study, we aimed to identify the protein cargo of leptospiral EVs released from intact leptospires grown under three different conditions: EMJH medium at 30 °C, temperature shifted to 37 °C, and physiologic osmolarity (EMJH medium with 120 mM NaCl). The naturally released EVs observed under transmission electron microscopy were spherical in shape with an approximate diameter of 80–100 nm. Quantitative proteomics and bioinformatic analysis indicated that the EVs were formed primarily from the outer membrane and the cytoplasm. The main functional COG categories of proteins carried in leptospiral EVs might be involved in cell growth, survival and adaptation, and pathogenicity. Relative to their abundance in EVs grown in EMJH medium at 30 °C, 39 and 69 proteins exhibited significant changes in response to the temperature shift and the osmotic change, respectively. During exposure to both stresses, *Leptospira* secreted several multifunctional proteins via EVs, while preserving certain virulence proteins within whole cells. Therefore, leptospiral EVs may serve as a decoy structure for host responses, whereas some virulence factors necessary for direct interaction with the host environment are reserved in leptospiral cells. This knowledge will be useful for understanding the pathogenesis of leptospirosis and developing as one of vaccine platforms against leptospirosis in the future.

## Introduction

Pathogenic *Leptospira* is the causative agent of leptospirosis, a common zoonotic disease worldwide^1^. Leptospirosis is considered a re-emerging disease with a high incidence in tropical and subtropical regions^1,2^. The number of leptospirosis cases has been underestimated due to its nonspecific clinical manifestations and the lack of readily available diagnostic tests. As a result, leptospirosis is considered one of the neglected tropical diseases^2^. Humans are accidental hosts and become infected by exposure to pathogenic leptospires shed through the urine of reservoir hosts, primarily rodents, into the environment, including water or soil^3^. Pathogenic leptospires enter the host through breaks in the skin or mucous membranes and then spread through the bloodstream to the target organs^4^.

Extracellular vesicles (EVs) are nanosized spherical proteolipids that are released from cells into the extracellular space to mediate intercellular communication^5^. EVs often carry multiple active molecules as cargoes, such as proteins, lipopolysaccharides (LPS), nucleic acids (DNA and/or RNA), and metabolites^5,6^. The release of EVs in bacteria can be triggered in response to hostile host environments, such as hydrogen peroxide, sodium chloride, antibiotics, and temperature shift^7–10^. EVs are known to contain outer membrane proteins (OMPs) and also carry secreted virulence factors, serving as a mechanism of bacterial secretion to transfer biological molecules to the host cells^11,12^. These bacterial EVs deliver a variety of virulence factors to reach target cells at both local and distant sites and play a role in bacterial pathogenesis^13^. For example, EVs secreted by pathogenic Escherichia coli carry Shiga toxin 2a (Stx2a), which induces cell death in colon epithelial cells^14^. For example, EVs secreted by pathogenic *Escherichia coli* carry Shiga toxin 2a (Stx2a), which induces cell death in colon epithelial cells^14^. EVs of *Bacteroides fragilis* deliver mature toxins to target cells and induce host cell injury^6^. EVs of *Legionella pneumophila* promote intracellular bacterial survival in macrophages^15^.

Pathogenic *Leptospira* have been shown to produce a structure similar to EVs, previously called ‘outer membrane vesicles’ (OMVs)^16–18^. The vesicles prepared by treating leptospires with alkaline plasmolysis buffer or citrate buffer contained multiple proteins with various functions and subcellular localizations^16–18^. However, the biogenesis and composition of leptospiral OMVs have not been proven to be derived only from the outer membrane (OM). Therefore, it would be more appropriate to refer to them generally as ‘extracellular vesicles’^19^. So far, the protein composition of EVs naturally released from intact *Leptospira* has never been reported. In addition, exogenous stress can influence the biogenesis and components of bacterial EVs^9,20^. Thus, proteomics of EVs produced under physiologic conditions would likely contribute to elucidating the pathophysiological functions of bacterial EVs in vivo. This study aimed to characterize the proteome of native leptospiral EVs produced in response to stress conditions that simulate the in vivo condition, including temperature shifted to 37 °C and physiologic osmolarity. The knowledge gained from this study should reveal the potential role of EVs in the pathogenesis of leptospirosis.

## Results

### Characterization of native leptospiral EVs

**Figure 1:**
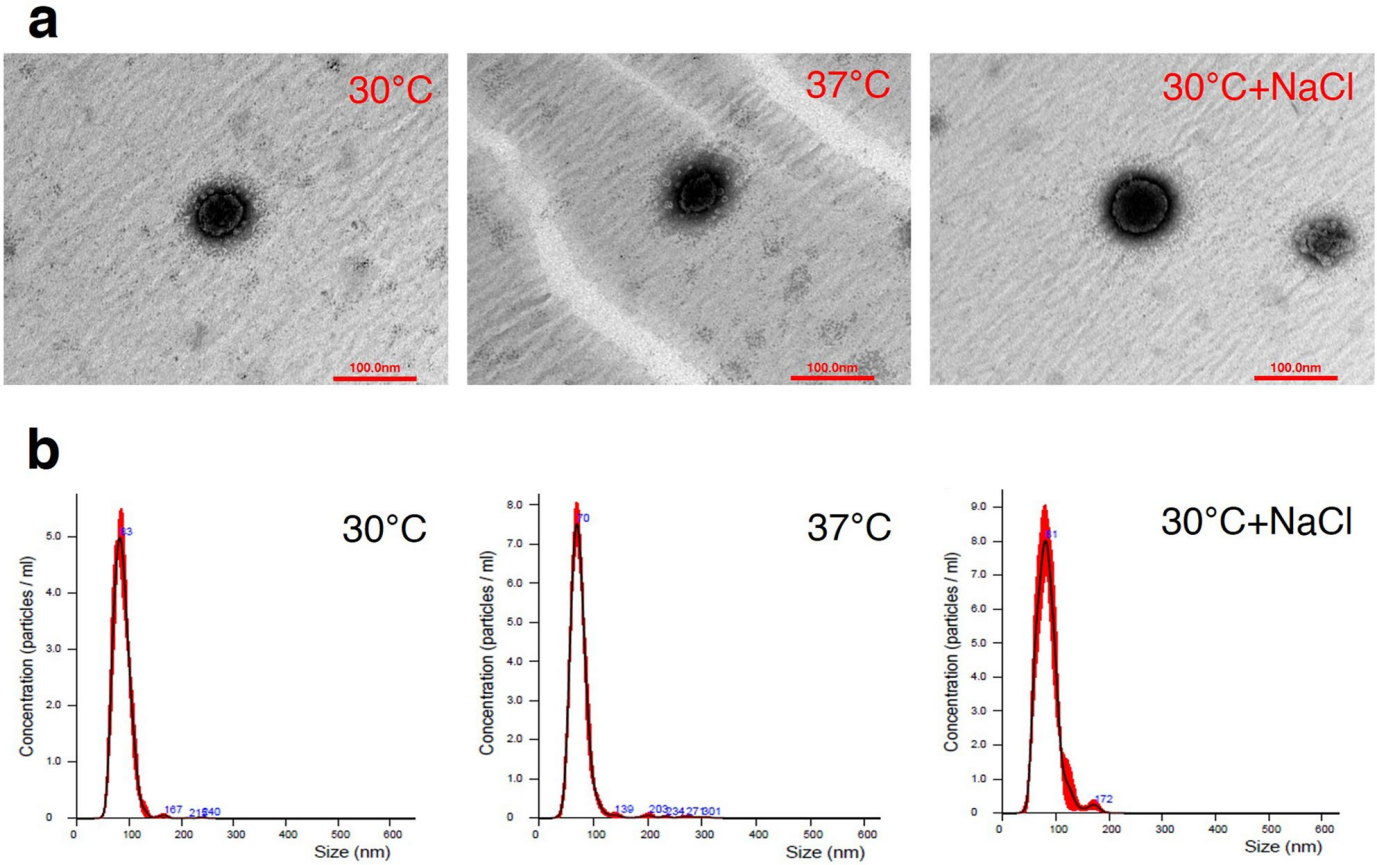
Characterization of native EVs from *Leptospira interrogans* serovar Pomona exposed to three culture conditions; in vitro EMJH medium at 30 °C, temperature shifted to 37 °C, and physiologic osmolarity at 30 °C with added 120 mM NaCl. Six biological replicates of each culture condition were performed. After sucrose gradient centrifugation, the enriched EVs in the selected sucrose fractions were pooled before characterization using transmission electron microscopy with negative staining (**a**), and dynamic light scattering (**b**). A, the EV samples were stained with UranyLess EM Stain and observed under JEM 1400 transmission electron microscope. B, the particle size distribution was measured using NanoSight NS300. The results represent mean of triplicate measurement with standard deviation of each culture condition. These figures are representative of the results obtained from the six biological replicates.

This study aimed to investigate EVs naturally released from intact viable leptospires. EVs in the culture supernatant were separated from intact leptospiral cells by centrifugation. The membrane integrity of leptospires in the cell pellets were evaluated using Live/Dead fluorescence staining. Most leptospiral cells derived from three conditions, including EMJH medium at 30 °C, temperature shifted to 37 °C, and physiologic osmolarity (EMJH medium plus 120 mM NaCl), were stained green with SYTO9 (Supplementary Fig. S1). This finding indicates that the naturally released EVs mainly originated from intact leptospires. To increase the purity of EVs, the sucrose density gradient centrifugation was performed. The putative density zones of the EVs varied from sucrose fractions 5–8 (Supplementary Fig. S2). Western blotting used to detect LipL32, a known major leptospiral outer membrane lipoprotein and likely a protein component of EVs showed the highest intensity of LipL32 in sucrose fractions 5–8 of all samples (Supplementary Fig. S3). Therefore, EVs were expected to be enriched in the fractions 5–8 and then were pooled before further analysis of their morphology and size distribution. TEM imaging revealed that the EVs had spherical shapes and an average diameter of about 80–100 nm in all conditions (Fig. 1a). The nanoparticle tracking analysis showed the homogeneous size distribution of leptospiral EVs from each condition (Fig. 1b). The average size of the EVs from the in vitro EMJH culture at 30 °C, the temperature shift, and the physiologic osmolarity were 86 ± 16, 77 ± 17, and 83 ± 15 nm in diameter, respectively. Moreover, the average concentrations of EVs were 3.06 ± 1.77 × 10^9^, 3.44 ± 2.60 × 10^9^, and 3.69 ± 2.77 × 10^9^ particles/ml for each condition, respectively, exhibiting no significant differences. Therefore, the detected variations of proteins under different conditions were attributed primarily to the differential expression of proteins within the EVs rather than to variations in the production of secreted EVs.

### Identification of proteins in native leptospiral EVs

**Figure 2:**
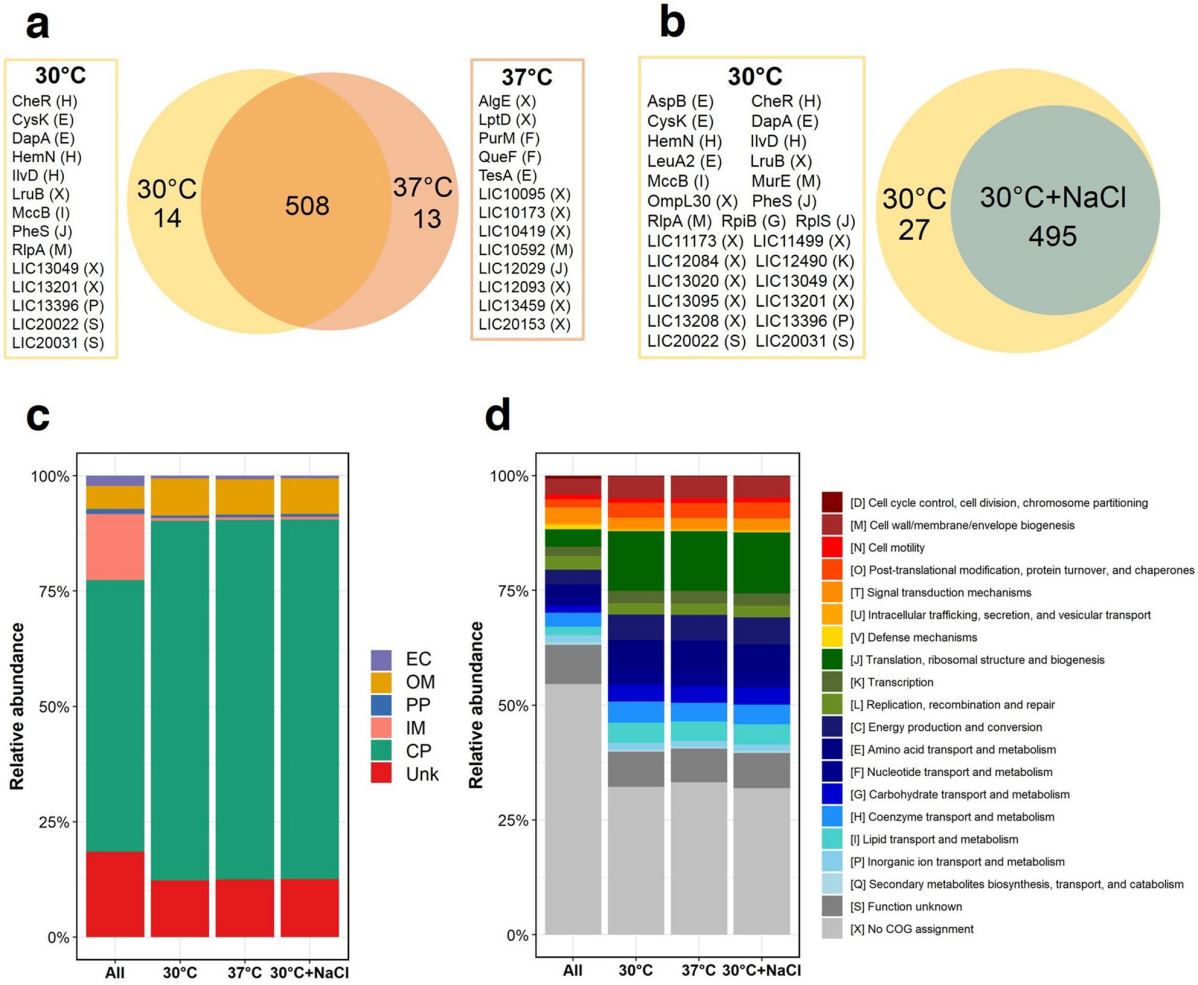
Identification of proteins in native EVs from *Leptospira interrogans* serovar Pomona exposed to three culture conditions; in vitro EMJH medium at 30 °C, temperature shifted to 37 °C, and physiologic osmolarity at 30 °C with added 120 mM NaCl. Six biological replicates of each culture condition were performed. The enriched EVs from the selected sucrose fractions were pooled before protein identification using LC–MS/MS. The mass spectrum data were analyzed using MaxQuant software. Data were searched against the protein database of *Leptospira interrogans* serovar Copenhageni Fiocruz L1-130. The proteins repeatedly identified by at least 3 out of 6 replicates were further analyzed. The number of total identified proteins in EVs produced during culture in EMJH medium at 30 °C was compared with 37 °C (**a**) or physiologic osmolarity (EMJH medium plus 120 mM NaCl) (**b**). Unique proteins in each condition were listed in the boxes and the predicted COG functional categories are shown in brackets. The predicted subcellular localizations (**c**) and COG functional categories (**d**) of the identified proteins and all theoretical proteins (All) were compared. C, subcellular localization includes extracellular (EC), outer membrane (OM), periplasm (PP), inner membrane (IM), cytoplasm (CP), and unknown (Unk).

The protein composition of isolated native EVs was characterized using LC–MS/MS. The MS data were then analyzed using MaxQuant software to identify proteins. The analysis revealed that 522, 521, and 495 proteins were present in EVs released from leptospires after culture in EMJH medium at 30 °C, exposure to a temperature shift, and osmotic change, respectively (Supplementary Data S1). A comparison of EV proteins from leptospires cultured at 30 °C and those challenged with a temperature change to 37 °C showed that 508 proteins were in common, while 14 and 13 proteins were unique to EVs released after incubation at 30 °C and 37 °C, respectively (Fig. 2a and Supplementary Table S1). At 30 °C, 495 proteins were detected in EVs produced under hypotonic (EMJH medium) and physiological (EMJH medium plus 120 mM NaCl) osmolarities. However, 27 proteins were exclusively detected in EVs from the hypotonic EMJH culture (Fig. 2b and Supplementary Table S2).

The subcellular localization patterns of proteins in the EV samples were found to be similar under the three different conditions (Fig. 2c). Based on the Clusters of Orthologous Groups (COGs), the identified 354, 348, and 337 EV proteins produced during culture at 30 °C in EMJH, exposure to the temperature shift and physiologic osmolarity, respectively, were classified into 19 identical functional categories (Fig. 2d). The COG distribution profiles were similar among all EV samples.

### Absolute quantification of proteins in native leptospiral EVs

**Figure 3:**
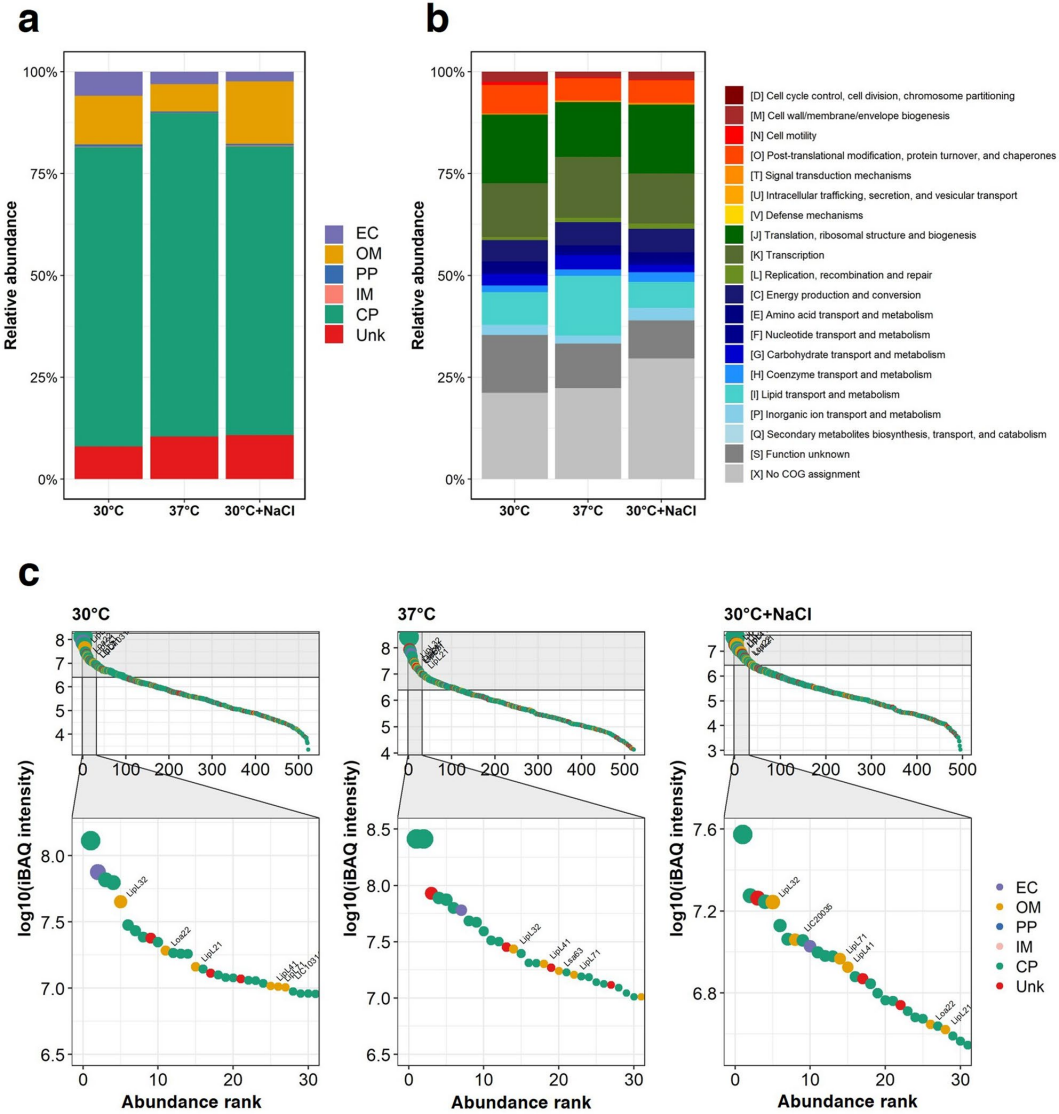
Absolute quantification of proteins in native EVs from *Leptospira interrogans* serovar Pomona exposed to three culture conditions; in vitro EMJH medium at 30 °C, temperature shifted to 37 °C, and physiologic osmolarity at 30 °C with added 120 mM NaCl. Six biological replicates of each culture condition were performed. The proteins repeatedly identified by at least 3 out of 6 replicates were further analyzed. The proteins identified in the EVs were quantitated using intensity-based absolute quantification (iBAQ). The sum of iBAQ values for each subcellular compartment (**a**) and COG functional category (**b**) were compared. The 30 most abundant proteins in the EVs obtained from each culture condition are shown (**c**). A and C, subcellular localization includes extracellular (EC), outer membrane (OM), periplasm (PP), inner membrane (IM), cytoplasm (CP), and unknown (Unk).

The molar quantities of identified EV proteins were determined using intensity-based absolute quantification (iBAQ) (Supplementary Data S1). The iBAQ values based on subcellular localizations were used to estimate the proportional abundance of membrane proteins in EVs. Total iBAQ values were compared for each subcellular compartment (Fig. 3a). The most abundant compartment of proteins in EVs produced under in vitro EMJH culture and physiologic osmolarity was the cytoplasm (CP, 70.7% and 72.0%), followed by the outer membrane (OM, 11.9% and 15.0%), unknown (Unk, 9.1% and 8.8%), extracellular (EC, 6.0% and 2.4%), periplasm (PP, 2.3% and 1.5%) and inner membrane (IM, 0.1% and 0.2%). The distribution pattern of the iBAQ values in EVs from the temperature shift was similar to the other two conditions, with the exception that OM (6.6%) was less abundant than Unk (11.4%). The distribution patterns of the COG categories based on the summed iBAQ values varied slightly depending on the culture conditions (Fig. 3b). However, the most prevalent COG categories in all EV samples were similar, including translation, ribosomal structure and biogenesis (J), function unknown (S), transcription (K), lipid transport and metabolism (I), post-translational modification, protein turnover, and chaperones (O), and energy production and conversion (C).

Moreover, the thirty most abundant proteins found in the EVs of each condition were displayed (Fig. 3c and Supplementary Table S3). Among these, several OMPs such as LipL21, LipL32, LipL41, LipL71, Loa22, Lsa63, and LIC20035, along with exoenzymes such as catalase (KatE) and hemolysin (LIC12631), were highly abundant. LipL32, LipL41, LipL71, and LIC12631 were identified as overlapping proteins, with LipL32 being the most abundant OMP.

### Relative quantification of proteins in native leptospiral EVs in response to temperature shift

**Figure 4:**
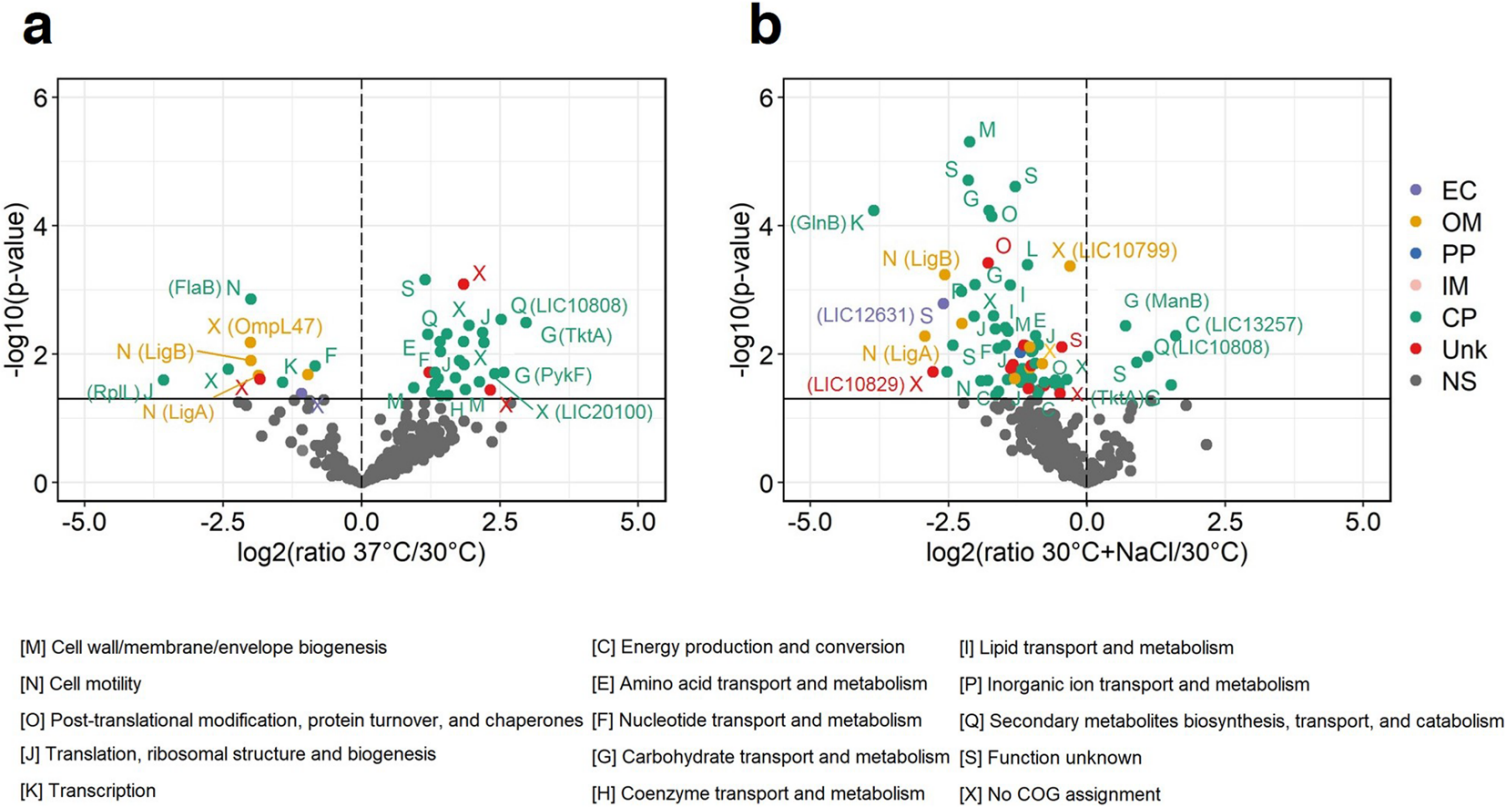
Relative quantification of proteins in native EVs from *Leptospira interrogans* serovar Pomona exposed to stress conditions; temperature shifted to 37 °C and physiologic osmolarity at 30 °C with added 120 mM NaCl compared with culture in EMJH medium at 30 °C. Six biological replicates of each culture condition were performed. The proteins repeatedly identified by at least 3 out of 6 replicates were further analyzed. The volcano plots show the distribution of the identified proteins in EVs according to -log10 (p-value) on the y-axis and log2 ratio on the x-axis. The log2 ratio is the comparison between temperature shift (37 °C/30 °C) **(a)** or osmolarity shift (30 °C + 120 mM NaCl/30 °C) **(b)** and culture at 30 °C. The log2 ratio of each comparison was compared with a value of 0, which represents no change (log2 of 1). The significant difference was calculated using the Student's *t*-test. Each dot represents a protein. Letters next to the dots are the predicted COG functional category and the protein names are shown in brackets. A and B, the predicted subcellular localization includes extracellular (EC), outer membrane (OM), periplasm (PP), inner membrane (IM), cytoplasm (CP), unknown (Unk), and not significant (NS).

Stable isotope dimethyl labeling was employed to quantitatively examine the relative changes in the protein abundance of EVs produced by leptospires when cultured under different conditions. When comparing incubations at 30 °C and 37 °C, a total of 352 proteins were differentially abundant (Supplementary Data S1). Of these, 39 proteins showed significant changes in abundance, with a bias towards the increased abundance of proteins in response to the temperature shift (28 of 39 proteins) (Fig. 4a and Supplementary Table S4). Most of the proteins with increased abundance were located in CP (25 of 28 proteins), and their COG functions were mainly related to cell wall/membrane/envelope biogenesis (M); carbohydrate transport and metabolism (G); translation, ribosomal structure and biogenesis (J); and secondary metabolites biosynthesis, transport and catabolism (Q). Five proteins with the highest increase in abundance were TktA, PykF, LIC10808, LIC20100, and LIC10984. Proteins with decreased abundance consisted of 5 proteins localized in CP, 4 proteins in OM, and one in EP or Unk location. Most of these proteins were related to cell motility (N). Proteins with the highest decrease in abundance were RplL, followed by LIC11499, OmpL47, FlaB, LigB, and LigA, which were significantly reduced in abundance by more than 1.8 times after temperature was shifted to 37 °C.

### Relative quantification of proteins in native leptospiral EVs in response to physiologic osmolarity

In comparison to the hypotonic EMJH condition, a total of 332 EV proteins showed altered abundance in response to osmotic change (Supplementary Data S1). Of these, 69 proteins demonstrated significant changes in abundance, with a strong bias toward the proteins with decreased abundance (64 out of 69 proteins) (Fig. 4b and Supplementary Table S5). However, five proteins located in CP, including ManB, TktA, LIC10808, LIC13240, and LIC13257, showed increased abundance. The COG annotation revealed that they were classified into the categories of energy production and conversion (C); carbohydrate transport and metabolism (G); and secondary metabolites biosynthesis, transport, and (Q). The majority of proteins with decreased abundance (44 out of 64) were located in CP. They belonged to 13 different functional COG categories such as post-translational modification, protein turnover, and chaperones (O); translation, ribosomal structure, and biogenesis (J); carbohydrate transport and metabolism (G); energy production and conversion (C); lipid transport and metabolism (I); and cell wall/membrane/envelope biogenesis (M). The top 5 proteins with the lowest relative abundance (more than 2.5-fold decrease) in response to physiologic osmolarity include GlnB, LigA, LigB, LIC10829, and LIC12631. Furthermore, several known virulence-associated proteins such as ClpA, FlaB1, GlnA, GroEL, LruC, OmpL36, OmpL47, LIC12324, and LIC12730 significantly decreased in response to the osmolarity shift.

## Discussion

Bacterial EVs serve as vehicles for delivering bacterial components and protecting them from harsh environments in the extracellular milieu, contributing to enhanced fitness and pathogenicity, and facilitate cell–cell or host-microbe interactions^5,21^. The mechanisms of leptospiral EV biogenesis and function remain unclear. Most previous studies on leptospiral EVs were chemically induced EVs^16–18^, which were formed by chemical treatments that caused cell lysis^17,18^. However, chemically induced EVs may have characteristics and protein composition that are different from those of naturally released EVs, possibly leading to inaccurate conclusions about the roles of EVs in the pathogenesis of leptospirosis. The temperature shift^22^ and the physiologic or isotonic osmolarity^23^, mimicking the host environment during infection, have been used to study protein expression in whole leptospiral cells but not in leptospiral EVs. This study characterized and compared leptospiral EVs that were naturally released after a temperature shifted to 37 °C and at physiologic osmolarity with those produced during in vitro growth in hypotonic EMJH medium at 30 °C to better predict the role of EVs in vivo.

Isolation of native EVs, excluding non-vesicular contaminants, such as residual proteins from lysed cells, is important to identify the actual protein composition of EVs^24,25^. In this study, leptospires were first harvested by low-speed centrifugation to minimize cell lysis, followed by filtration to remove large contaminants. However, cell lysis is unavoidable because the cell membrane of *Leptospira* is fragile and easily damaged^26^. Therefore, the sucrose density gradient centrifugation based on the buoyant density of the macromolecule was used to purify EVs^24,25^ including leptospiral EVs^16–18^. The EVs in the pooled samples of the enriched fractions had a good homogeneity of the particle size distribution (~ 80 nm) (Fig. 1b) and a typical spherical shape (Fig. 1a), similar to EVs produced by other bacteria^27,28^, suggesting that leptospiral EVs were successfully purified. In contrast to the narrow zone of chemically induced leptospiral EVs after sucrose gradient ultracentrifugation^16,18^, leptospiral EVs in this study were located in multiple fractions. The difference in the density zone width might be due to the amount of particles or EVs in individual sample because a high concentration of particles can increase the zone width after sucrose gradient centrifugation^29^. However, the spreading of zones of minor components contaminated in the sample cannot be excluded^30^. Thus, we performed six biological replicates of each culture condition to ensure reproducible results and gave precedence to commonly found proteins (at least 3 out of 6 samples) with high abundance.

Quantification of total proteins in the EV preparation as a means of normalization could be problematic because the protein content and composition could vary widely between preparations of EVs derived under different conditions^7^. Therefore, we used the iBAQ measurement to estimate the absolute molar quantities of the identified proteins and the proportional abundance of each subcellular localization. Although the majority of high-abundance proteins in leptospiral EVs were likely from the cytoplasm, the molar abundance of proteins from the outer membrane was remarkably higher than that from the periplasm and the inner membrane (Fig. 3a). These findings suggest that native leptospiral EVs are likely to originate from the outer membrane and simultaneously carry a group of cytoplasmic proteins. Therefore, it should be correct to call leptospiral EVs “leptospiral outer membrane vesicles”^19^. The abundance of proteins identified in native EVs under all three conditions was mainly in the functional COG categories involved in the transcription and translation processes (K and J), lipid transport and metabolism (I), energy production (C), and post-translational modification, protein turnover, and chaperones (O) (Fig. 3b), which are probably needed for cellular processes, information storage, and metabolism in bacterial cells. The marginal alterations in COGs of differentially expressed proteins (Fig. 2d) might be a result of the scant changes of proteins within the EVs in response to a single stress condition, most of which were in the same COGs. However, when using iBAQ to calculate the proportional abundance of each COG category, the COG profile exhibited slight variations depending on the culture conditions (Fig. 3b).

In principle, bacterial EVs can promote cell survival by transporting unwanted or harmful molecules to the extracellular milieu to preserve their life and resources^5^. Like other bacterial pathogens^31^, leptospires may release certain metabolic proteins normally located in the cytoplasm into the external environment to maintain the optimal cell homeostasis. Moreover, several proteins of *Leptospira* have functional redundancy, for example, EF-Tu and GroEL, which are classified into transcription and translation processes, and post-translational modification, respectively^32–34^. The multifunctional roles of these proteins in pathogenicity have been reported^32,33^. EVs carrying these proteins might contribute to their moonlighting functions. In addition, leptospiral EVs contain known virulence-associated proteins, for example, EF-Tu, LigA, LigB, LipL21, LipL32, LipL41, LipL46, LipL71, Loa22, Lsa26, Lsa63, OmpL1 and Sph1 (Supplementary Data S1). The interaction of Lig, LipL21, or OmpL1 proteins to fibrinogen has been shown to inhibit fibrin clot formation^35–38^. The dispersal of EVs harboring these proteins could potentially restrict the extent of fibrin clot formation in the vicinity of leptospires, thereby facilitating bacterial dissemination and contributing to hemorrhage. EF-Tu, LipL32, LipL41, LipL46, and OmpL1 were shown to bind to plasminogen^32,38–41^. Plasminogen bound to EVs might be converted to plasmin, which could degrade host proteins such as fibronectin and fibrinogen and activate host matrix metalloproteases, resulting in tissue damage^32,38–41^. Loa22 has been shown to directly induce cytotoxicity and inflammation in a rat proximal tubule cell line^42^, suggesting that EVs carrying this protein might be involved in leptospiral nephropathy. In a mouse model of chronic infection, membrane vesicles along with aggregated leptospires were observed to adhere to the brush borders of proximal renal tubular epithelial cells, probably resulting in the formation of a biofilm-like structure to promote long-term colonization^43^. In addition, certain proteins on EVs may play a defensive role during infection by sequestering antibodies and acting as decoy antigens to divert the attention of the immune system. Therefore, leptospiral EVs could play an important role in the pathogenesis of leptospirosis.

The unique and differentially abundant proteins of EVs produced in response to distinct stress conditions suggest that leptospiral EVs may have specific adaptive responses to each condition. Leptospires secreted 13 unique proteins, and 28 significantly increased proteins via EVs in response to the temperature change to 37 °C (Figs. 2a and 4a). Among these, PykF and TktA in the carbohydrate transport (G) category, as well as LIC10808 and LIC13206 in the secondary metabolites biosynthesis, transport, and catabolism (Q) category, showed an exclusive response to the temperature shift. Pyruvate kinase (Pyk) regulates carbohydrate metabolism by catalyzing the biosynthesis of pyruvate and ATP from phosphoenolpyruvate. Pyruvate kinase isoenzymes are typically involved in pyruvate synthesis and cell growth in most bacteria^44,45^. As a potent scavenger of H_2_O_2,_ pyruvate has previously been shown to protect pathogenic leptospires from H_2_O_2_ killing^46^. Therefore, exogenous pyruvate in EVs might ameliorate oxidative stress against pathogenic leptospires during infection. In this study, three transketolase encoding proteins, Tkt, TktA, and TktC, were secreted in EVs with increased abundance. Transketolase catalyzes several key reactions of the non-oxidative branches of the pentose phosphate pathway. This enzyme served as a virulence and/or fitness factor in several bacterial pathogens^47,48^. In response to physiologic osmolarity, five proteins increased significantly in EVs (Fig. 4b). Four out of five proteins were annotated as metabolic enzymes, such as NADH oxidoreductase, transketolase, and phosphomannomutase (PMM). The PMM encoded by *manB* catalyzes the conversion of mannose-6-phosphate to mannose-1-phosphate, which is subsequently converted to GDP-mannose. GDP-mannose is consumed in the biosynthesis of various macromolecular polymers^49–52^ to build bacterial structures. These findings suggest that EVs may play a role in the growth, survival, and adaptation of leptospires in response to temperature and osmotic stresses. The vesicles might offer some protection against stress-inducing conditions.

Interestingly, several virulence factors and virulence-associated proteins were underrepresented in EVs after the temperature and/or osmotic shift (Figs. 2 and 4), for example, ClpB, FlaB, GlnA, GroEL, LigA, LigB, LruB, LruC, Sph2 or MFn4, OmpL30, OmpL47, RlpA, and LIC12730. There are possible explanations for these findings, including: (1) the expression of these proteins may be induced by other stress signals that were not tested in this study, (2) they may be secreted by other mechanisms or secretion systems that are not related to EVs, and (3) they may be important for the function of intact leptospires, resulting in low abundance in EVs. The vesiculation process may be an adaptive response that selectively removes unwanted proteins while retaining proteins that are required for direct interaction with neighboring cells or the host environment in intact leptospiral cells. This process enables bacteria to survive and adapt to their environment. A similar study on the leptospiral exoproteome using label-free quantification demonstrated the protein abundance in whole cells and the culture supernatant of *L. interrogans* serovar Manilae exposed to the temperature shifted to 37 °C and isotonic osmolarity^34^. However, the EVs were not isolated and purified in that study; therefore, the exoproteome could contain proteins both in secreted soluble form and naturally released EVs. They showed that ClpB, FlaB, GlnA, GroEL, LigA, LruC, OmpL47, and LIC20197 were more abundant in whole cells than in the culture supernatant, and LigB and LIC12730 were dominant in whole cells, suggesting that these proteins remained in leptospiral cells. On the contrary, the expression of LruB was detected only in the culture supernatant; thus, it could be secreted either as soluble exoproteins or in EVs. In both stress conditions, Sph2, OmpL30, and RlpA proteins were low or undetectable in the exoproteome, similar to our finding in EVs. However, these protein levels were also low in whole cells, suggesting that their expression might be suppressed during exposure to these stress conditions.

Notably, we found OppA, a homolog of the surface-associated protein of *Treponema denticola*, to be of particular interest. OppA protein was present in low abundance in EVs released by leptospires during growing in vitro conditions and highly increased abundance in response to the temperature shift (Supplementary Data S1). OppA has been reported to bind soluble plasminogen and fibronectin but does not participate in bacterial adherence to cell-bound receptors^53,54^. While membrane vesicles have been reported in oral spirochetes, including *T. denticola*, the presence and function of OppA in EVs has not been studied. Further investigations are required to elucidate its role in pathogenic *Leptospira* and leptospiral EVs.

This study has some limitations. We focused only on the protein composition of EVs produced under particular host environments in response to temperature shift and physiologic osmolarity. Additional investigations are required to verify the roles of EVs in protecting leptospires against stress-inducing conditions, including host immune responses. Other types of cargo, such as DNA and RNA, that are packaged into EVs upon stress need to be further studied. In addition, the bioinformatic prediction tools available and utilized in this study, which were primarily developed based on subsets of proteins from model organisms and may not fully align proteins exhibiting lower sequence identity to the datasets, such as those found in spirochetes, including *Leptospira*. Several proteins in leptospiral EVs are categorized either under unknown functions (S category) or remain undesignated to any COG categories (X category), rendering their roles inconclusive. Consequently, if a significant number of differentially expressed proteins belong to these categories, minimal changes are likely to be observed across all conditions.

In conclusion, this study characterized native leptospiral EVs released from intact leptospires during in vitro cultivation and exposure to the temperature shift or physiologic osmolarity. Quantitative proteomics and bioinformatic analyses suggested that the biogenesis of native leptospiral EVs was mainly from the outer membrane and the cytoplasm. The proteins identified in the EVs might be involved in bacterial adaptation and pathogenicity during *Leptospira* infection under physiologic environments. In response to stress conditions, leptospires secreted several multifunctional or moonlighting proteins through EVs but preserved certain virulence and virulence-associated proteins in their cells. Therefore, the native leptospiral EVs might serve as an external decoy for the host immune responses, while virulence-associated proteins crucial for direct interaction with host components might be restored in the whole cell structure. This proteomic analysis of native leptospiral EVs showed the changes in their cargo, suggesting the functional advantages and adaptive response to cells grown in in vivo-simulated environments. The knowledge will be useful for better understanding of the pathogenesis of leptospirosis. In addition, EVs have been tested as one of vaccine platforms for leptospirosis^17^, therefore our results should be crucial for the development of better vaccines against leptospirosis in the future.

## Methods

### Leptospiral cultivation and stress conditions

This study utilized a low-passage (< 5 in vitro passage) isolate of *Leptospira interrogans* serovar Pomona, which was isolated from infected hamsters obtained from our previous study^55^. Leptospires were grown in Ellinghausen-McCullough-Johnson-Harris (EMJH) medium containing *Leptospira* Medium Base EMJH (BD Difco™, USA) with 10% (v/v) bovine serum albumin (BSA) supplement solution^56^ at 30 °C until the exponential phase was reached (~ 2 × 10^8^ cells/ml). Approximately 1 × 10^8^ cells of leptospires in exponential phase were initially grown at 30 °C overnight before exposure to stress conditions. To simulate the conditions encountered by *Leptospira* during infection, the temperature was shifted from 30 °C to 37 °C overnight, as previously described^22^. To mimic the physiologic osmolarity, leptospires were cultured in EMJH medium supplemented with 120 mM NaCl at 30 °C overnight, as previously described^23^. For in vitro growth condition, the overnight culture was incubated at 30 °C overnight. Six biological replicates of each culture condition were performed.

### Isolation and purification of leptospiral EVs

The isolation and purification processes were designed based on methods for gram-negative bacteria^24,25^. Intact leptospiral cells were removed by centrifugation at 3000×*g* at 4 °C for 15 min and filtration through a 0.22 µm nitrocellulose membrane (Merck Millipore, Ireland). The cell membrane disruption of the cell pellets was determined using the Live/Dead Bac Light Bacterial Viability Kit (Thermo Scientific), as previously described^57^. Leptospiral cells were treated with cold absolute methanol for 5 min on ice and used as a non-intact cell control^57^. The supernatant was centrifuged at 200,000×*g* at 4 °C for 1 h. The pellets were collected and resuspended with BSA-free *Leptospira* Medium Base EMJH solution.

Leptospiral EVs were purified by sucrose density gradient centrifugation as previously described^16–18^ with some modifications. The stepwise sucrose density gradient was prepared by gently pipetting down 800 µl of each tris sodium chloride buffer (50 mM Tris and 100 mM NaCl) containing 5% increasing sucrose concentrations from 20 to 60% (w/v). The sample (800 µl) was added on top of the sucrose gradient, followed by centrifugation at 77,000×*g* at 4 °C overnight. The entire gradient was separated into 10 fractions (800 µl each) by gentle pipetting from the top of the gradient. Protein concentrations were measured using Micro BCA™ Protein Assay Kit (Thermo Scientific), following the manufacturer’s instruction.

### Sodium dodecyl sulfate polyacrylamide electrophoresis (SDS-PAGE) and Western blotting

The proteins (10 µl) were separated on 15% polyacrylamide gel and either stained with Coomassie Brilliant Blue R-250 (Bio-Rad, Germany) as previously described^58^ or transferred onto a nitrocellulose membrane. The Western blot for detection of LipL32 was performed as previously described^57^ with some modifications. Anti-LipL32 mouse monoclonal antibody (1:10,000, in-house preparation) and horseradish peroxidase (HRP)-conjugated goat anti-mouse IgG antibody (1:5000, KPL) were used as primary and secondary antibodies, respectively. The protein bands were detected with ECL chemiluminescent substrate (Amersham ECL Prime, GE Healthcare).

### Transmission electron microscopy (TEM)

The morphology of leptospiral EVs was determined by negative staining using UranyLess EM Stain (Delta Microscopies, France). The EV samples (10 µl) were gently placed on grids (Formvar/Carbon Coated-Copper 200 mesh, Polysciences) for 1 min at room temperature (RT). The grids were washed twice with sterile distilled water (DW) before placing them on a drop of 10 µl of UranyLess solution for 1 min at RT. The samples were dried in a desiccator for 5 min and observed with JEM 1400 transmission electron microscopy (Jeol Ltd., Japan).

### Nanoparticle tracking analysis

The size distribution of the leptospiral EVs was determined by nanoparticle tracking analysis using NanoSight NS300 (Malvern Panalytical, UK) coupled with NanoSight Software NTA. The EV samples were diluted 50 times with sterile DW. Each diluted sample was injected into the Low Volume Flow Cell chamber at a speed of 50 µl/s. Each biological replicate was analyzed in triplicate.

### Protein extraction and in-solution digestion

Leptospiral EVs were treated with lysis buffer (2% SDS in 100 mM triethyl ammonium bicarbonate, TEAB) and disrupted by sonication (amplitude 35%, pulse of 10 s, and rest of 5 s, for a total of 5 min). The protein samples were reduced with 10 mM DTT at 37 °C with mild agitation for 30 min and alkylated with 40 mM iodoacetamide at RT for 30 min in the dark. The reaction was quenched by incubation with 10 mM DTT at RT for 15 min. Protein samples were incubated with 6 volumes of cold acetone at − 20 °C overnight. Subsequently, the samples were centrifuged at 12,000×*g* at 4 °C for 10 min. The pellet was reconstituted with 0.6 M urea in TEAB buffer and then sonicated for 10 min. The samples were digested with porcine trypsin (Thermo Scientific) in a 1:50 (w/w) ratio at 37 °C overnight. Finally, the peptide samples were completely dried in vacuo. The peptide concentrations were measured using Quantitative Fluorometric Peptide Assay (Thermo Scientific), following the manufacturer’s instruction.

### Stable isotope dimethyl labeling

The digested peptides were isotopically labeled as previously described^59,60^ with some modifications. The EV samples from three different conditions: in vitro culture at 30 °C, temperature shifted to 37 °C, and physiologic osmolarity (six biological replicates per condition), were differentially labeled with light, medium, and heavy isotopes, respectively. The dried samples were reconstituted with TEAB buffer and incubated with 4% (v/v) CH_2_O, CD_2_O, or ^13^CD_2_O in formaldehyde solution for light, medium, or heavy labeling, respectively. The samples were further incubated with 0.6 M NaBH_3_CN for light and medium labeling or 0.6 M NaBD_3_CN for heavy labeling at RT with mild agitation for 1 h. The reactions were quenched by incubation with 1% ammonium and then with formic acid (FA). The labeled samples were pooled in a 1:1:1 (v/v/v) ratio and completely dried. Subsequently, the mixed labeled samples were separated into 10 fractions using the Pierce High pH Reversed-Phase Peptide Fractionation Kit (Thermo Scientific), following the manufacturer’s instruction. The elution solution contained 5% increasing concentrations of acetonitrile (ACN) from 5 to 50% (v/v) in 0.1% triethylamine. The eluted samples were completely dried and then reconstituted in 0.1% FA before applying them to LC–MS/MS.

### Liquid chromatography with tandem mass spectrometry (LC–MS/MS)

The peptide mixtures were analyzed by LC–MS/MS using an EASY-nLC1000 system coupled to a Q-Exactive Orbitrap Plus mass spectrometer equipped with a nanoelectrospray ion source (Thermo Scientific), as previously described^60^ with some modifications. Three hundred nanograms (5 µl) of the peptide mixture was applied to LC–MS/MS. The peptides were eluted with 5% to 20% ACN containing 0.1% FA for 45 min, followed by 40% to 98% ACN containing 0.1% FA for 10 min at a flow rate of 300 nl/min. The MS methods included a full MS scan at a mass resolution of 70,000, followed by 10 data-dependent MS2 scans at a resolution of 17,500. The normalized collision energy of HCD fragmentation was set at 27%. An MS scan range of 400 m/z to 1600 m/z was selected, and monoisotopic precursor ions with unassigned charge states with a charge state of + 1 or greater than + 8 were excluded. A dynamic exclusion of 30 s was used.

### Data analysis

Protein identification and quantification were performed using the MaxQuant software suite (version 2.1.1.0) with the Andromeda search engine. Raw MS files were searched against the complete sequence of *L. interrogans* serovar Copenhageni Fiocruz L1-130 protein retrieved from the UniProt database (www.uniprot.org, taxonomic identifier 267671). The default search parameters with some additional settings were used. The false discovery rate (FDR) of 0.01, Trypsin/P as a specific digesting enzyme with 2 missed cleavage, peptide tolerance first search of 20 ppm, and main search of 4.5 ppm were set. Cysteine carbamidomethylation and different dimethyl isotope labels of the N termini and lysine were set as fixed modifications. The oxidation of methionine was set as a variable modification. Any contaminants and decoy sequences were removed. The proteins repeatedly identified by at least 3 out of 6 replicates were considered proteins that consistently existed within leptospiral EVs. The label-free quantification (LFQ) and intensity-based absolute quantification (iBAQ) of peptide intensities were performed to calculate protein abundance. The iBAQ algorithm estimates the molar quantities of the proteins by dividing the raw protein intensities by the number of theoretically observable tryptic peptides. The default setting of LFQ and iBAQ with the “match between run” feature was employed. Dimethyl labeling was used to calculate the relative abundance of proteins. For label-based quantification, the monoisotopic mass increment for the light, medium, and heavy isotopes were set as 28.0313, 32.0564, and 36.0757 Da, respectively. The normalized ratios of heavy/light (H/L) isotopes and medium/light (M/L) isotopes (Supplementary Data S1) were used for relative quantitative analysis.

Subcellular localization was based on previously published experimental data (Supplementary Data S2) and bioinformatic prediction using PSORTb v3.0.3^61^, CELLO v.2.5^62^, and SOSUI-GramN^63^ with a majority voting strategy. Proteins that were predicted to be at different locations by each tool were assigned to unknown. The annotation of functions based on the Clusters of Orthologous Groups (COGs) category was searched using the eggNOG mapper web^64^. Proteins that were annotated with more than one functional category were ignored.

### Statistical analysis

The log2 normalized ratios of temperature shift/in vitro growth (37 °C/30 °C, M/L) or physiologic osmolarity/in vitro growth (30 °C + NaCl/30 °C, H/L) were compared with a value of 0 (no change, log2 of 1). The ratio found by at least 3 out of 6 replicates was statistically analyzed. The significant difference was calculated using Student's *t*-test in MS Excel. The p-value < 0.05 or the proteins detected in a single culture condition were considered statistically significant. The data were visualized using R studio.

### Supplementary Information

Supplementary Information 1.

Supplementary Information 2.

Supplementary Figure S1.

Supplementary Figure S2.

Supplementary Figure S3.

Supplementary Table S1.

Supplementary Table S2.

Supplementary Table S3.

Supplementary Table S4.

Supplementary Table S5.

## Data Availability

The mass spectrometry proteomics data have been deposited to the ProteomeXchange Consortium via the PRIDE partner repository with the dataset identifier PXD042648.
