# Supplementary material for: Proteomic profile of naturally released extracellular vesicles secreted from Leptospira interrogans serovar Pomona in response to temperature and osmotic stresses
Source: Sci Rep. 2023 Oct 30;13:18601. doi: 10.1038/s41598-023-45863-0 (PMC10616267; doi:10.1038/s41598-023-45863-0)
Supplement: Supplementary file 2 — Supplementary Information 2. [file 41598_2023_45863_MOESM2_ESM.docx]

List of experimentally verified leptospiral outer membrane proteins (OMPs) from previous reports. Relevant articles were retrieved from MEDLINE (PubMed) and Scopus databases. Information regarding the OMPs, including gene and protein IDs, as well as gene and protein names, was derived from the UniProt database ([www.uniprot.org](http://www.uniprot.org)) based on the complete sequence of *L. interrogans* serovar Copenhageni Fiocruz L1-130 protein (taxonomic identifier 267671).

| **Gene ID** | **Gene name** | **Protein ID** | **Protein name** | **Reference** |
| --- | --- | --- | --- | --- |
| LIC10009 | *lp25* | Q72WC8 | Putative lipoprotein | [^1^] |
| LIC10011 | *lipL21* | Q72WC6 | LipL21 | [^2^] |
| LIC10054 | *mpL36* | Q72W83 | Probable endolytic peptidoglycan transglycosylase | [^3^] |
| LIC10091 | *lipL40* | Q72W49 | Putative lipoprotein | [^3^] |
| LIC10191 | *loa22* | Q72VV5 | Peptidoglycan associated cytoplasmic membrane protein | [^4,5^] |
| LIC10258 | *ompA, lsa66* | Q72VN8 | OmpA-like domain-containing protein | [^6^] |
| LIC10314 | *lsa63* | Q72VI3 | Uncharacterized protein | [^3,7^] |
| LIC10368 | *lsa21* | Q72VD2 | Putative lipoprotein | [^8^] |
| LIC10464 | *ligB* | Q72V39 | Ig-like repeat domain protein 3 | [^9^] |
| LIC10465 | *ligA* | G1UB65 | Ig-like repeat domain protein 1 | [^9^] |
| LIC10486 | *pL40* | Q72V17 | Uncharacterized protein | [^10^] |
| LIC10494 | *lic10494* | Q72V09 | Putative lipoprotein | [^3^] |
| LIC10508 | *lic10508* | Q72UZ5 | Putative lipoprotein | [^11^] |
| LIC10509 | *lic10509* | Q72UZ4 | Putative lipoprotein | [^3^] |
| LIC10537 | *mfn9* | Q72UX0 | Peptidoglycan associated periplasmic protein | [^12^] |
| LIC10574 | *omp52* | Q72UT3 | Dihydroorotase | [^13^] |
| LIC10714 | *mfn2* | Q72UE3 | Outer membrane receptor protein | [^12^] |
| LIC10765 | *mpL17* | Q72U96 | Uncharacterized protein | [^3,14^] |
| LIC10774 | *lic10774* | Q72U87 | Putative lipoprotein | [^15^] |
| LIC10793 | *lp49* | Q72U69 | Antigen Lp49 | [^3^] |
| LIC10879 | *lsa16* | Q72TY5 | Putative lipoprotein | [^16^] |
| LIC10920 | *lsa24.9* | Q72TU6 | Putative lipoprotein | [^17^] |
| LIC10973 | *ompL1* | Q72TP4 | Outer membrane protein | [^18,19^] |
| LIC11003 | *lipL71* | Q72TL5 | LipL71 | [^20^] |
| LIC11009 | *lsa26* | Q72TK9 | Uncharacterized protein | [^21^] |
| LIC11030 | *lic11030* | Q72TJ1 | Putative lipoprotein | [^22^] |
| LIC11087 | *lsa30* | Q72TD4 | Putative lipoprotein | [^23^] |
| LIC11122 | *lsa19* | Q72T99 | Putative lipoprotein | [^24^] |
| LIC11360 | *lsa23* | Q72SL9 | Putative lipoprotein | [^21^] |
| LIC11436 | *mfn7* | Q72SE7 | FecR domain-containing protein | [^12^] |
| LIC11456 | *lipL31* | Q72SC8 | LipL31 | [^25^] |
| LIC11458 | *ostA* | Q72SC6 | Outer membrane protein, porin superfamily | [^26^] |
| LIC11469 | *lsa20* | Q72SB5 | Uncharacterized protein | [^22^] |
| LIC11570 | *gspD* | Q72S17 | General secretory pathway protein D | [^26^] |
| LIC11574 | *lic11574* | Q72S13 | Prepilin-type N-terminal cleavage/methylation domain-containing protein | [^11,27^] |
| LIC11612 | *mfn1* | Q72RX6 | Uncharacterized protein | [^12^] |
| LIC11623 | *bamA* | Q72RW5 | Outer membrane protein | [^26^] |
| LIC11711 | *lic11711* | Q72RN4 | Putative lipoprotein | [^28^] |
| LIC11834 | *lsa33* | Q72RB4 | Putative lipoprotein | [^29^] |
| LIC11848 | *ompL32* | Q72RA0 | Uncharacterized protein | [^30^] |
| LIC11851 | *impL63* | Q72R97 | Cytoplasmic membrane protein | [^25^] |
| LIC11885 | *lipL46* | Q72R63 | Putative lipoprotein | [^31^] |
| LIC11947 | *lcpA* | Q72R05 | Putative lipoprotein | [^32^] |
| LIC11975 | *lsa36* | Q72QY3 | Outer membrane protein | [^21^] |
| LIC11996 | *erpY-like* | Q72QY9 | Uncharacterized protein | [^33^] |
| LIC12238 | *lic12238* | Q72Q74 | Uncharacterized protein | [^3^] |
| LIC12253 | *lsa25* | Q72Q60 | Putative lipoprotein | [^29^] |
| LIC12254 | *omp85* | Q72Q59 | Outer membrane protein | [^26^] |
| LIC12263 | *ompL37* | Q72Q50 | Uncharacterized protein | [^18^] |
| LIC12287 | *lsa14* | Q72Q27 | Putative lipoprotein | [^24^] |
| LIC12307 | *tolC* | Q72Q08 | Outer membrane TolC superfamily | [^26^] |
| LIC12341 | *lic12341* | Q72PX6 | Toluene tolerance protein | [^11^] |
| LIC12587 | *lic12587* | Q72P88 | Putative lipoprotein | [^28^] |
| LIC12730 | *lic12730* | Q72NU9 | TPR-REGION domain-containing protein | [^3^] |
| LIC12875 | *tuf* | Q72NF9 | Elongation factor Tu (EF-Tu) | [^34^] |
| LIC12892 | *lp29* | Q72NE2 | Putative lipoprotein | [^3^] |
| LIC12895 | *lsa27* | Q72ND9 | Putative lipoprotein | [^3,35^] |
| LIC12906 | *lsa24* | Q72NC8 | Putative lipoprotein | [^36^] |
| LIC12922 | *lic12922* | Q72NB3 | PpiC domain-containing protein | [^3^] |
| LIC12966 | *lipL41* | Q72N71 | LipL41 | [^37^] |
| LIC13050 | *ompL47* | Q72MY9 | Uncharacterized protein | [^18^] |
| LIC13059 | *lsa25.6* | Q72MY0 | Putative lipoprotein | [^16^] |
| LIC13060 | *lipL36* | Q72MX9 | LipL36 | [^38^] |
| LIC13086 | *lic13086* | Q72MV3 | Putative lipoprotein | [^39^] |
| LIC13131 | *mpL21* | Q72MR2 | YceI domain-containing protein | [^3,14^] |
| LIC13143 | *tlyC* | Q72MQ0 | Hemolysin | [^40^] |
| LIC13166 | *ompL36* | Q72MM7 | OmpL36 | [^18^] |
| LIC13259 | *lic13259* | Q72MC9 | Putative lipoprotein | [^41^] |
| LIC13341 | *lic13341* | Q72M50 | Putative lipoprotein | [^42^] |
| LIC13491 | *ompL54* | Q72LR2 | Metallopeptidase | [^18^] |
| LIC13411 |  | Q72LY2 | Putative lipoprotein | [^11,27^] |
| LIC20035 | *lic20035* | Q75G29 | Uncharacterized protein | [^43^] |
| LIC20151 | *hbpA* | Q75FN1 | TonB-dependent outer membrane receptor | [^44^] |
| LIC20172 | *lruC* | Q75FL0 | Lipoprotein | [^45,46^] |

References

1 Abreu, P. A. E. *et al.* Lp25 membrane protein from pathogenic *Leptospira* spp. is associated with rhabdomyolysis and oliguric acute kidney injury in a guinea pig model of leptospirosis. *PLoS Negl Trop Dis* **11**, e0005615, doi:10.1371/journal.pntd.0005615 (2017).

2 Cullen, P. A., Haake, D. A., Bulach, D. M., Zuerner, R. L. & Adler, B. LipL21 is a novel surface-exposed lipoprotein of pathogenic *Leptospira* species. *Infect Immun* **71**, 2414-2421, doi:10.1128/iai.71.5.2414-2421.2003 (2003).

3 Vieira, M. L. *et al.* In vitro identification of novel plasminogen-binding receptors of the pathogen *Leptospira interrogans*. *PLoS One* **5**, e11259, doi:10.1371/journal.pone.0011259 (2010).

4 Koizumi, N. & Watanabe, H. Molecular cloning and characterization of a novel leptospiral lipoprotein with OmpA domain. *FEMS Microbiol Lett* **226**, 215-219, doi:10.1016/S0378-1097(03)00619-0 (2003).

5 Ristow, P. *et al.* The OmpA-like protein Loa22 is essential for leptospiral virulence. *PLoS Pathog* **3**, e97, doi:10.1371/journal.ppat.0030097 (2007).

6 Oliveira, R. *et al.* Characterization of novel OmpA-like protein of *Leptospira interrogans* that binds extracellular matrix molecules and plasminogen. *PLoS One* **6**, e21962, doi:10.1371/journal.pone.0021962 (2011).

7 Vieira, M. L. *et al.* Lsa63, a newly identified surface protein of *Leptospira interrogans* binds laminin and collagen IV. *J Infect* **60**, 52-64, doi:10.1016/j.jinf.2009.10.047 (2010).

8 Atzingen, M. V. *et al.* Lsa21, a novel leptospiral protein binding adhesive matrix molecules and present during human infection. *BMC Microbiol* **8**, 70, doi:10.1186/1471-2180-8-70 (2008).

9 Matsunaga, J. *et al.* Pathogenic *Leptospira* species express surface-exposed proteins belonging to the bacterial immunoglobulin superfamily. *Mol Microbiol* **49**, 929-945, doi:10.1046/j.1365-2958.2003.03619.x (2003).

10 Zhao, W. *et al.* Molecular characterization of the pL40 protein in *Leptospira interrogans*. *Can J Microbiol* **55**, 739-749, doi:10.1139/w09-014 (2009).

11 Evangelista, K. V. *et al.* Identification of cell-binding adhesins of *Leptospira interrogans*. *PLoS Negl Trop Dis* **8**, e3215, doi:10.1371/journal.pntd.0003215 (2014).

12 Pinne, M., Matsunaga, J. & Haake, D. A. Leptospiral outer membrane protein microarray, a novel approach to identification of host ligand-binding proteins. *J Bacteriol* **194**, 6074-6087, doi:10.1128/JB.01119-12 (2012).

13 Hsieh, W. J., Chang, Y. F., Chen, C. S. & Pan, M. J. Omp52 is a growth-phase-regulated outer membrane protein of *Leptospira santarosai* serovar Shermani. *FEMS Microbiol Lett* **243**, 339-345, doi:10.1016/j.femsle.2004.12.021 (2005).

14 Oliveira, T. R. *et al.* Evaluation of leptospiral recombinant antigens MPL17 and MPL21 for serological diagnosis of leptospirosis by enzyme-linked immunosorbent assays. *Clin Vaccine Immunol* **15**, 1715-1722, doi:10.1128/cvi.00214-08 (2008).

15 Passalia, F. J., Carvalho, E., Heinemann, M. B., Vieira, M. L. & Nascimento, A. The *Leptospira interrogans* LIC10774 is a multifunctional surface protein that binds calcium and interacts with host components. *Microbiol Res* **235**, 126470, doi:10.1016/j.micres.2020.126470 (2020).

16 Pereira, P. R. M. *et al.* Multifunctional and redundant roles of *Leptospira interrogans* proteins in bacterial-adhesion and fibrin clotting inhibition. *Int J Med Microbiol* **307**, 297-310, doi:10.1016/j.ijmm.2017.05.006 (2017).

17 Rossini, A. D. *et al.* Identification of a novel protein in the genome sequences of *Leptospira interrogans* with the ability to interact with host's components. *J Microbiol Immunol Infect* **53**, 163-175, doi:10.1016/j.jmii.2018.12.012 (2020).

18 Pinne, M. & Haake, D. A. A comprehensive approach to identification of surface-exposed, outer membrane-spanning proteins of Leptospira interrogans. *PLoS One* **4**, e6071, doi:10.1371/journal.pone.0006071 (2009).

19 Haake, D. A. *et al.* Molecular cloning and sequence analysis of the gene encoding OmpL1, a transmembrane outer membrane protein of pathogenic *Leptospira* spp. *J Bacteriol* **175**, 4225-4234, doi:10.1128/jb.175.13.4225-4234.1993 (1993).

20 Zhang, K. *et al.* Leptospiral LruA is required for virulence and modulates an interaction with mammalian apolipoprotein AI. *Infect Immun* **81**, 3872-3879, doi:10.1128/iai.01195-12 (2013).

21 Siqueira, G. H. *et al.* Characterization of three novel adhesins of *Leptospira interrogans*. *Am J Trop Med Hyg* **89**, 1103-1116, doi:10.4269/ajtmh.13-0205 (2013).

22 Mendes, R. S. *et al.* The novel leptospiral surface adhesin Lsa20 binds laminin and human plasminogen and is probably expressed during infection. *Infect Immun* **79**, 4657-4667, doi:10.1128/iai.05583-11 (2011).

23 Souza, N. M. *et al.* Lsa30, a novel adhesin of *Leptospira interrogans* binds human plasminogen and the complement regulator C4bp. *Microb Pathog* **53**, 125-134, doi:10.1016/j.micpath.2012.06.001 (2012).

24 Figueredo, J. M. *et al.* Characterization of two new putative adhesins of *Leptospira interrogans*. *Microbiology (Reading)* **163**, 37-51, doi:10.1099/mic.0.000411 (2017).

25 Haake, D. A. & Matsunaga, J. Characterization of the leptospiral outer membrane and description of three novel leptospiral membrane proteins. *Infect Immun* **70**, 4936-4945, doi:10.1128/IAI.70.9.4936-4945.2002 (2002).

26 Haake, D. A. & Matsunaga, J. *Leptospira*: a spirochaete with a hybrid outer membrane. *Mol Microbiol* **77**, 805-814, doi:10.1111/j.1365-2958.2010.07262.x (2010).

27 Surdel, M. C., Hahn, B. L., Anderson, P. N. & Coburn, J. Heterologous production of the adhesin LIC13411 from pathogenic *Leptospira* facilitates binding of non-pathogenic *Leptospira* in vitro and in vivo. *Front Cell Infect Microbiol* **12**, 917963, doi:10.3389/fcimb.2022.917963 (2022).

28 Kochi, L. T. *et al.* The interaction of two novel putative proteins of *Leptospira interrogans* with E-cadherin, plasminogen and complement components with potential role in bacterial infection. *Virulence* **10**, 734-753, doi:10.1080/21505594.2019.1650613 (2019).

29 Domingos, R. F. *et al.* “Features of two proteins of *Leptospira interrogans* with potential role in host-pathogen interactions”. *BMC Microbiology* **12**, 50, doi:10.1186/1471-2180-12-50 (2012).

30 Eshghi, A. *et al.* Methylation and in vivo expression of the surface-exposed *Leptospira interrogans* outer-membrane protein OmpL32. *Microbiology (Reading)* **158**, 622-635, doi:10.1099/mic.0.054767-0 (2012).

31 Matsunaga, J., Werneid, K., Zuerner, R. L., Frank, A. & Haake, D. A. LipL46 is a novel surface-exposed lipoprotein expressed during leptospiral dissemination in the mammalian host. *Microbiology (Reading)* **152**, 3777-3786, doi:10.1099/mic.0.29162-0 (2006).

32 Barbosa, A. S. *et al.* Functional characterization of LcpA, a surface-exposed protein of *Leptospira* spp. that binds the human complement regulator C4BP. *Infect Immun* **78**, 3207-3216, doi:10.1128/iai.00279-10 (2010).

33 Ghosh, K. K. *et al.* Role of supramolecule Erpy-like lipoprotein of *leptospira* in thrombin-catalyzed fibrin clot inhibition and binding to complement factors h and i, and its diagnostic potential. *Infect Immun* **87**, doi:10.1128/iai.00536-19 (2019).

34 Wolff, D. G. *et al.* Interaction of *Leptospira* elongation factor Tu with plasminogen and complement factor H: a metabolic leptospiral protein with moonlighting activities. *PLoS One* **8**, e81818, doi:10.1371/journal.pone.0081818 (2013).

35 Longhi, M. T. *et al.* A newly identified protein of *Leptospira interrogans* mediates binding to laminin. *J Med Microbiol* **58**, 1275-1282, doi:10.1099/jmm.0.011916-0 (2009).

36 Barbosa, A. S. *et al.* A newly identified leptospiral adhesin mediates attachment to laminin. *Infect Immun* **74**, 6356-6364, doi:10.1128/iai.00460-06 (2006).

37 Shang, E. S., Summers, T. A. & Haake, D. A. Molecular cloning and sequence analysis of the gene encoding LipL41, a surface-exposed lipoprotein of pathogenic *Leptospira* species. *Infect Immun* **64**, 2322-2330, doi:10.1128/iai.64.6.2322-2330.1996 (1996).

38 Haake, D. A. *et al.* Characterization of leptospiral outer membrane lipoprotein LipL36: downregulation associated with late-log-phase growth and mammalian infection. *Infect Immun* **66**, 1579-1587, doi:10.1128/iai.66.4.1579-1587.1998 (1998).

39 Passalia, F. J., Heinemann, M. B., Vieira, M. L. & Nascimento, A. A novel *Leptospira interrogans* protein LIC13086 inhibits fibrin clot formation and interacts with host components. *Front Cell Infect Microbiol* **11**, 708739, doi:10.3389/fcimb.2021.708739 (2021).

40 Carvalho, E. *et al.* Leptospiral TlyC is an extracellular matrix-binding protein and does not present hemolysin activity. *FEBS Lett* **583**, 1381-1385, doi:10.1016/j.febslet.2009.03.050 (2009).

41 Cavenague, M. F. *et al.* Characterization of a novel protein of *Leptospira interrogans* exhibiting plasminogen, vitronectin and complement binding properties. *Int J Med Microbiol* **309**, 116-129, doi:10.1016/j.ijmm.2018.12.005 (2019).

42 Ghosh, K. K., Prakash, A., Shrivastav, P., Balamurugan, V. & Kumar, M. Evaluation of a novel outer membrane surface-exposed protein, LIC13341 of *Leptospira*, as an adhesin and serodiagnostic candidate marker for leptospirosis. *Microbiology (Reading)* **164**, 1023-1037, doi:10.1099/mic.0.000685 (2018).

43 Ghosh, K. K., Prakash, A., Balamurugan, V. & Kumar, M. Catecholamine-modulated novel surface-exposed adhesin LIC20035 of *leptospira* spp. Binds host extracellular matrix components and is recognized by the host during infection. *Appl Environ Microbiol* **84**, doi:10.1128/aem.02360-17 (2018).

44 Asuthkar, S., Velineni, S., Stadlmann, J., Altmann, F. & Sritharan, M. Expression and characterization of an iron-regulated hemin-binding protein, HbpA, from *Leptospira interrogans* serovar Lai. *Infect Immun* **75**, 4582-4591, doi:10.1128/iai.00324-07 (2007).

45 Verma, A. *et al.* Antibodies to a novel leptospiral protein, LruC, in the eye fluids and sera of horses with *Leptospira*-associated uveitis. *Clin Vaccine Immunol* **19**, 452-456, doi:10.1128/CVI.05524-11 (2012).

46 Toma, C. *et al.* Leptospiral outer membrane protein LMB216 is involved in enhancement of phagocytic uptake by macrophages. *Cell Microbiol* **16**, 1366-1377, doi:10.1111/cmi.12296 (2014).
