# Supplementary material for: Proteomic profile of naturally released extracellular vesicles secreted from Leptospira interrogans serovar Pomona in response to temperature and osmotic stresses
Source: Sci Rep. 2023 Oct 30;13:18601. doi: 10.1038/s41598-023-45863-0 (PMC10616267; doi:10.1038/s41598-023-45863-0)
Supplement: Supplementary file 3 — Supplementary Figure S1. [file 41598_2023_45863_MOESM3_ESM.docx]

**
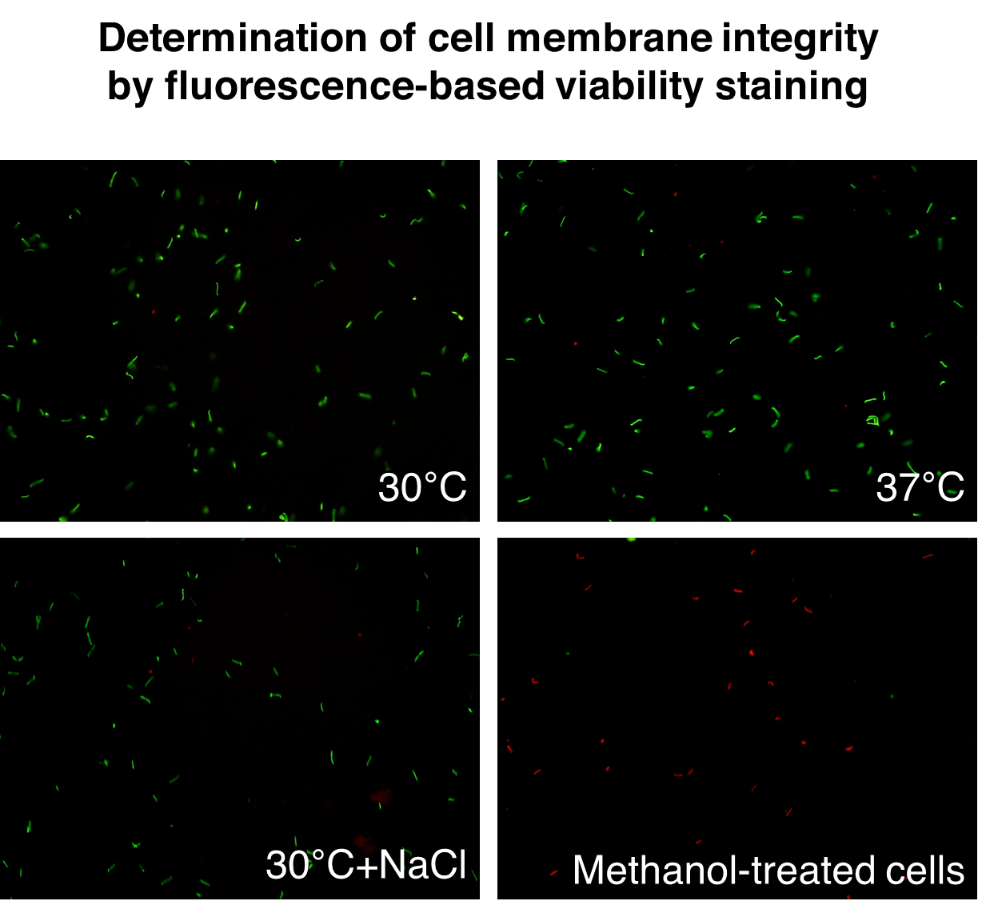
**

**Figure S1** The membrane integrity of leptospires from three culture conditions; in vitro EMJH medium at 30 °C (30°C), temperature shifted to 37 °C (37°C), and physiological osmolarity at 30 °C with 120 mM NaCl added (30°C+NaCl). Approximately 1 × 10^8^ cells of leptospires were initially grown at 30 °C overnight before exposure to stresses; temperature change, and physiologic osmolarity, or further incubation at 30 °C overnight. Six biological replicates of each culture condition were performed. Intact leptospiral cells were removed by centrifugation at 3,000 × g at 4 °C for 15 min. The integrity of the leptospiral cell membrane in the cell pellets was determined using Live/Dead fluorescence staining. Methanol-treated leptospiral cells were used to represent non-intact cells. The stained cells were observed under a fluorescence microscope at 400× magnification. Green (SYTO9) and red (PI) staining indicates intact cells and cells with damaged membranes, respectively. The figures are representative of the results obtained from the six biological replicates. Most leptospires grown under three conditions were stained green with SYTO9, while control cells of non-intact leptospires were stained red with PI.
