# Supplementary material for: Proteomic profile of naturally released extracellular vesicles secreted from Leptospira interrogans serovar Pomona in response to temperature and osmotic stresses
Source: Sci Rep. 2023 Oct 30;13:18601. doi: 10.1038/s41598-023-45863-0 (PMC10616267; doi:10.1038/s41598-023-45863-0)
Supplement: Supplementary file 4 — Supplementary Figure S2. [file 41598_2023_45863_MOESM4_ESM.docx]

**
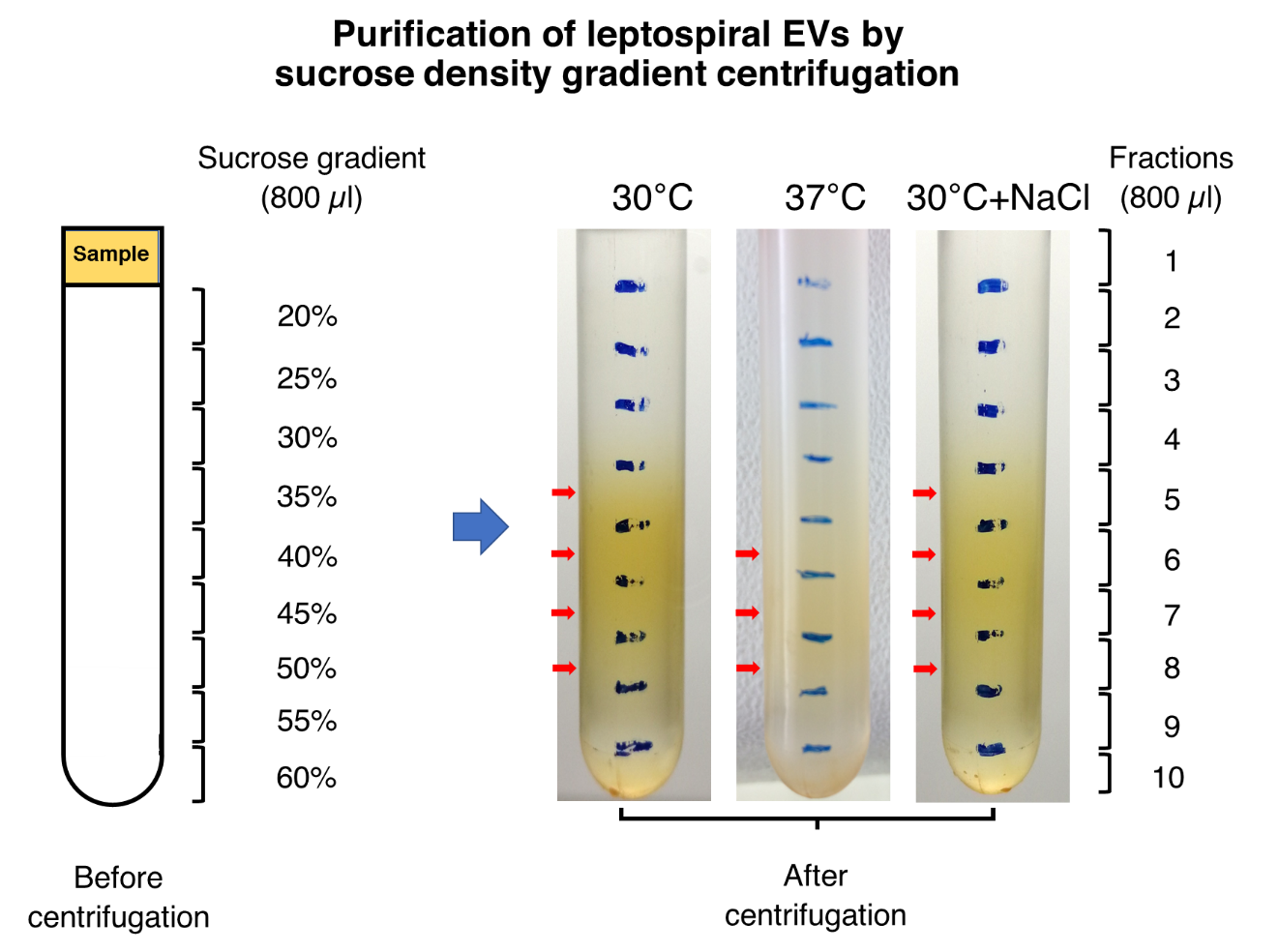
**

**Figure S2** Purification of native leptospiral extracellular vesicles (EVs). The native EVs were isolated from leptospire culture in EMJH medium at 30 °C (30°C), temperature shifted to 37 °C (37°C), and physiologic osmolarity by addition of 120 mM NaCl (30°C+NaCl). Six biological replicates of each culture condition were performed. The culture supernatants were initially centrifuged at 3,000 × g at 4 °C for 15 min, filtration through a 0.22 µm nitrocellulose membrane, and finally centrifuged at 200,000 × g at 4 °C for 1 h. The pellets were collected and resuspended with BSA-free *Leptospira* Medium Base EMJH solution. The density sucrose gradient was prepared by gently pipetting down tris sodium chloride buffer containing 5% increasing sucrose concentrations from 20% to 60% (w/v). The sample (800 µl) was added onto the top of the sucrose gradient followed by centrifugation at 77,000 × g at 4 °C overnight. The entire gradient was separated into 10 fractions (800 µl each) by pipetting from the top of the gradient. The figures are representative of the results obtained from the six biological replicates. The putative density zones of the EVs from incubation at 30 °C and physiologic osmolarity were visible in yellow at sucrose fractions 5−8, while those from temperature shift were found in fractions 6−8 (red arrow).
