# Supplementary material for: Proteomic profile of naturally released extracellular vesicles secreted from Leptospira interrogans serovar Pomona in response to temperature and osmotic stresses
Source: Sci Rep. 2023 Oct 30;13:18601. doi: 10.1038/s41598-023-45863-0 (PMC10616267; doi:10.1038/s41598-023-45863-0)
Supplement: Supplementary file 5 — Supplementary Figure S3. [file 41598_2023_45863_MOESM5_ESM.docx]

**
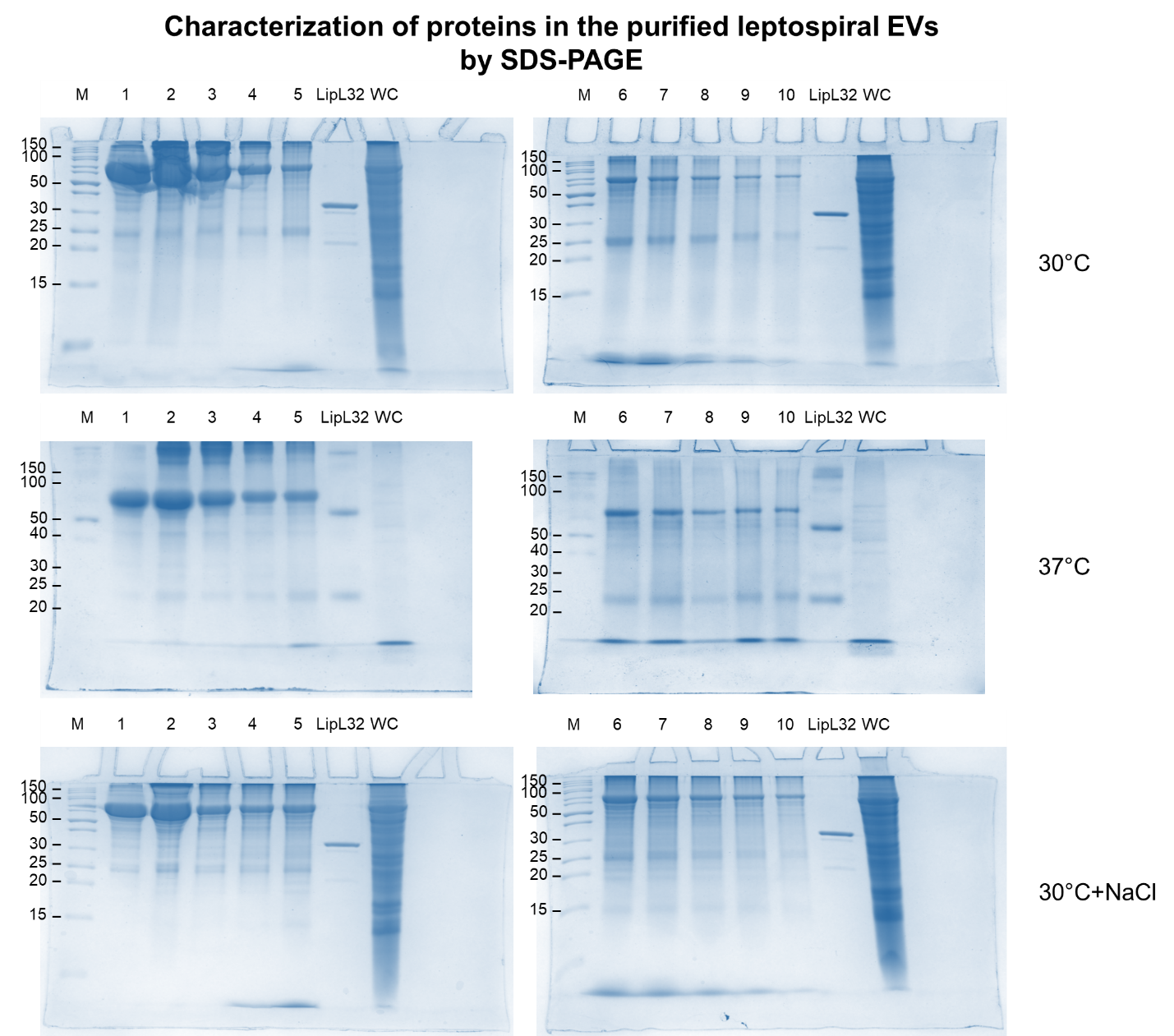
**

**
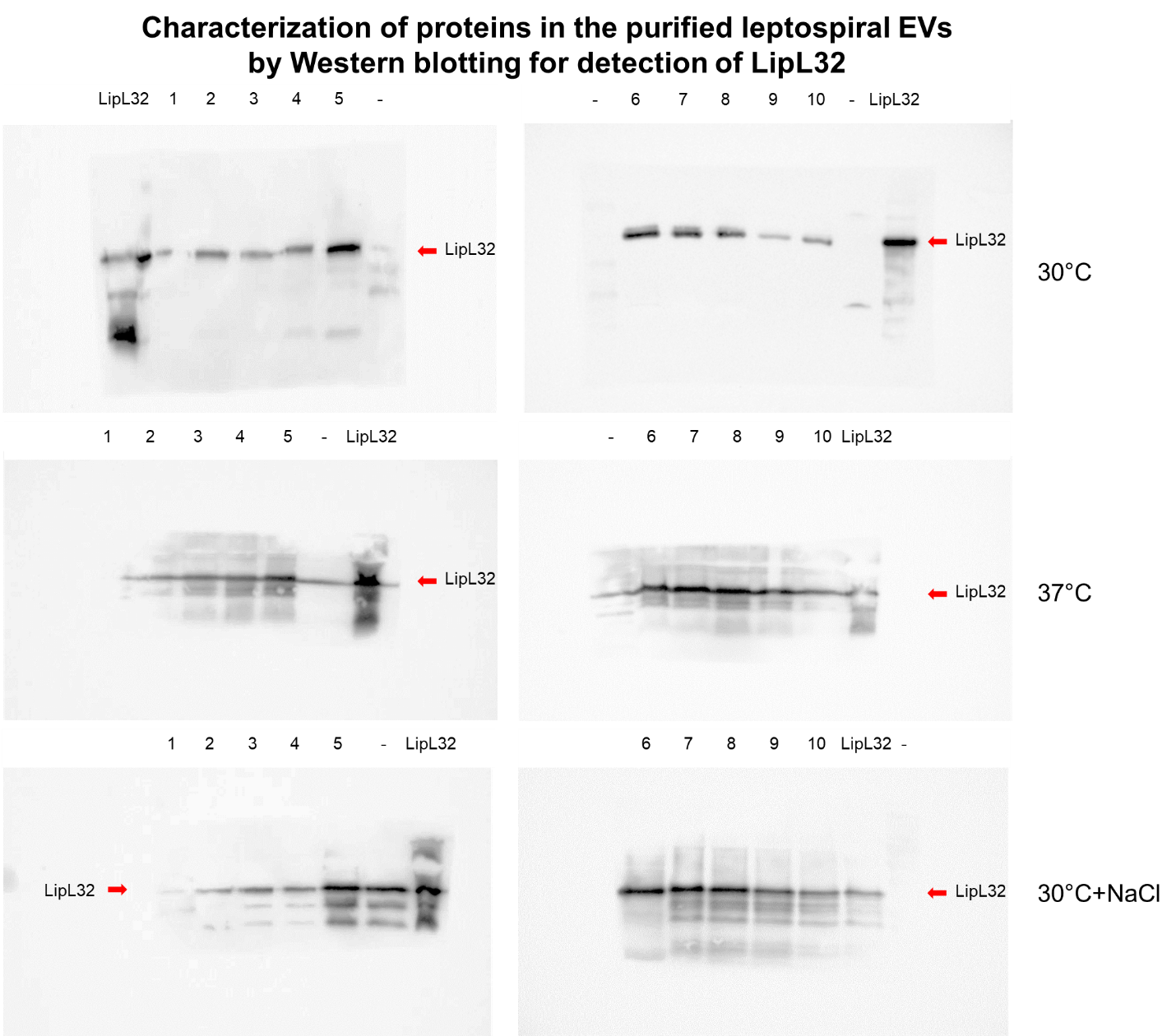
**

**Figure S3** Characterization of purified native leptospiral extracellular vesicles (EVs). The native EVs were isolated from leptospire culture in EMJH medium at 30 °C (30°C), temperature shifted to 37 °C (37°C), and physiologic osmolarity by addition of 120 mM NaCl (30°C+NaCl). Six biological replicates of each culture condition were performed. The culture supernatants were initially centrifuged at 3,000 × g at 4 °C for 15 min, filtration through a 0.22 µm nitrocellulose membrane, and finally centrifuged at 200,000 × g at 4 °C for 1 h. The pellets were collected, resuspended with BSA-free *Leptospira* Medium Base EMJH solution, and purified using sucrose density gradient centrifugation. The density sucrose gradient was prepared by gently pipetting down tris sodium chloride buffer containing 5% increasing sucrose concentrations from 20% to 60% (w/v). The sample (800 µl) was added onto the top of the sucrose gradient followed by centrifugation at 77,000 × g at 4 °C overnight. The entire gradient was separated into 10 fractions (800 µl each) by pipetting from the top of the gradient. The proteins of purified EVs in each sucrose fraction were characterized by 15% SDS-PAGE and either Coomassie Brilliant Blue R-250 staining or Western blotting for detection of LipL32. Anti-LipL32 mouse monoclonal antibody (1:10,000, in-house preparation) and horseradish peroxidase (HRP)-conjugated goat anti-rabbit IgG antibody (1:5,000, KPL) were used as primary and secondary antibodies, respectively. The protein bands in immunoblot membranes were detected with ECL chemiluminescent substrate (Amersham ECL Prime, GE Healthcare). These figures are representative of the results obtained from the six biological replicates. The position of PageRuler Unstained Protein Ladder (Thermo Scientific) is indicated to the left of the molecular weight marker lane (M). The protein samples from sucrose fractions 1 – 10 are denoted by the numbers above the figure, while the recombinant LipL32 is represented by LipL32. The lanes marked by WC in SDS-PAGE gels are leptospiral whole cell lysate. However, the lanes marked by (-) in Western blot membranes are empty and may have been contaminated by protein samples from adjacent lanes. The Western blotting showed the highest intensity of LipL32 in fractions 5−8 of all samples.
