# Supplementary material for: Proteomic profile of naturally released extracellular vesicles secreted from Leptospira interrogans serovar Pomona in response to temperature and osmotic stresses
Source: Sci Rep. 2023 Oct 30;13:18601. doi: 10.1038/s41598-023-45863-0 (PMC10616267; doi:10.1038/s41598-023-45863-0)
Supplement: Supplementary file 6 — Supplementary Table S1. [file 41598_2023_45863_MOESM6_ESM.docx]

**S1 Table.** The list of unique proteins in leptospiral EVs released following incubation at 30 °C compared with 37 °C.

| **Gene Names** | **Protein IDs** | **Protein names** | **Unique condition** | **Subcellular localization** | **COG** |
| --- | --- | --- | --- | --- | --- |
| *lic13459* | Q72LU0 | Zn-Tnp-IS91 domain-containing protein | 37 °C | Cytoplasm | X |
| *algE* | Q72LX6 | Alginate-exp domain-containing protein | 37 °C | Outer membrane | X |
| *lic12093* | Q72QM0 | Carb-bd dom fam9 domain-containing protein | 37 °C | Cytoplasm | X |
| *lic12029* | Q72QT0 | tRNA/rRNA methyltransferase | 37 °C | Cytoplasm | J |
| *queF* | Q72RB6 | NADPH-dependent 7-cyano-7-deazaguanine reductase | 37 °C | Cytoplasm | F |
| *lptD* | Q72SC6 | Outer membrane protein, porin superfamily | 37 °C | Outer membrane | X |
| *tesA* | Q72SG8 | Acyl-CoA thioesterase | 37 °C | Unknown | E |
| *purM* | Q72T98 | Phosphoribosylformylglycinamidine cyclo-ligase | 37 °C | Cytoplasm | F |
| *lic10592* | Q72UR5 | Peptidoglycan-associated Cytoplasm membrane protein | 37 °C | Cytoplasm | M |
| *lic10419* | Q72V82 | WYL domain-containing protein | 37 °C | Cytoplasm | X |
| *lic10173* | Q72VX2 | Uncharacterized protein | 37 °C | Unknown | X |
| *lic10095* | Q72W45 | Secreted protein | 37 °C | Cytoplasm | X |
| *lic20153* | Q75FM9 | Lipoprotein | 37 °C | Extracellular | X |
| *lic13396* | Q72LZ7 | Flavin-containing monooxygenase 5 | 30 °C | Cytoplasm | P |
| *pheS* | Q72M80 | Phenylalanine-tRNA ligase alpha subunit | 30 °C | Cytoplasm | J |
| *lic13201* | Q72MI6 | Adenylate/guanylate cyclase | 30 °C | Cytoplasm | X |
| *lic13049* | Q72MZ0 | Flagellar filament outer layer protein FlaA domain protein | 30 °C | Cytoplasm | X |
| *cysK* | Q72QN1 | Cysteine synthase | 30 °C | Cytoplasm | E |
| *cheR* | Q72R77 | Protein-glutamate O-methyltransferase | 30 °C | Cytoplasm | H |
| *ilvD* | Q72TC0 | Dihydroxy-acid dehydratase | 30 °C | Cytoplasm | H |
| *dapA* | Q72U22 | 4-hydroxy-tetrahydrodipicolinate synthase | 30 °C | Cytoplasm | E |
| *lruB* | Q72UE4 | Putative lipoprotein | 30 °C | Outer membrane | X |
| *mccB* | Q72V59 | 3-methylcrotonoyl-CoA carboxylase beta subunit | 30 °C | Cytoplasm | I |
| *rlpA* | Q72W83 | Probable endolytic peptidoglycan transglycosylase | 30 °C | Outer membrane | M |
| *hemN* | Q75G00 | Coproporphyrinogen-III oxidase | 30 °C | Cytoplasm | H |
| *lic20031* | Q75G14 | Short-chain dehydrogenase | 30 °C | Outer membrane | S |
| *lic20022* | Q75G27 | Azoreductase | 30 °C | Cytoplasm | S |
