# Supplementary material for: Proteomic profile of naturally released extracellular vesicles secreted from Leptospira interrogans serovar Pomona in response to temperature and osmotic stresses
Source: Sci Rep. 2023 Oct 30;13:18601. doi: 10.1038/s41598-023-45863-0 (PMC10616267; doi:10.1038/s41598-023-45863-0)
Supplement: Supplementary file 7 — Supplementary Table S2. [file 41598_2023_45863_MOESM7_ESM.docx]

**S2 Table.** The list of unique proteins in leptospiral EVs released following cultivation in EMJH medium compared with EMJH medium supplemented with 120 mM NaCl.

| **Gene Names** | **Protein IDs** | **Protein names** | **Unique condition** | **Subcellular localization** | **COG** |
| --- | --- | --- | --- | --- | --- |
| *lic13396* | Q72LZ7 | Flavin-containing monooxygenase 5 | EMJH | Cytoplasm | P |
| *pheS* | Q72M80 | Phenylalanine-tRNA ligase alpha subunit | EMJH | Cytoplasm | J |
| *lic13208* | Q72MH9 | Riboflavin-specific deaminase/reductase | EMJH | Cytoplasm | X |
| *lic13201* | Q72MI6 | Adenylate/guanylate cyclase | EMJH | Cytoplasm | X |
| *lic13095* | Q72MU4 | Tetratricopeptide repeat protein | EMJH | Cytoplasm | X |
| *lic13049* | Q72MZ0 | Flagellar filament outer layer protein FlaA domain protein | EMJH | Cytoplasm | X |
| *lic13020* | Q72N18 | VWA domain-containing protein | EMJH | Cytoplasm | X |
| *lic12490* | Q72PI1 | RNA polymerase sigma factor | EMJH | Cytoplasm | K |
| *aspB* | Q72PP6 | Aminotransferase | EMJH | Cytoplasm | E |
| *lic12084* | Q72QM9 | NAD-binding-3 domain-containing protein | EMJH | Cytoplasm | X |
| *cysK* | Q72QN1 | Cysteine synthase | EMJH | Cytoplasm | E |
| *cheR* | Q72R77 | Protein-glutamate O-methyltransferase | EMJH | Cytoplasm | H |
| *murE* | Q72R81 | UDP-N-acetylmuramoyl-L-alanyl-D-glutamate-2,6-diaminopimelate ligase | EMJH | Cytoplasm | M |
| *leuA2* | Q72RZ1 | 2-isopropylmalate synthase 2 | EMJH | Cytoplasm | E |
| *rplS* | Q72S27 | 50S ribosomal protein L19 | EMJH | Cytoplasm | J |
| *lic11499* | Q72S85 | DUF1931 domain-containing protein | EMJH | Cytoplasm | X |
| *ompL30* | Q72SV1 | OMP-b-brl domain-containing protein | EMJH | Outer membrane | X |
| *lic11173* | Q72T48 | PF03961 family protein | EMJH | Cytoplasm | X |
| *ilvD* | Q72TC0 | Dihydroxy-acid dehydratase (DAD) | EMJH | Cytoplasm | H |
| *dapA* | Q72U22 | 4-hydroxy-tetrahydrodipicolinate synthase (HTPA synthase) | EMJH | Cytoplasm | E |
| *lruB* | Q72UE4 | Putative lipoprotein | EMJH | Outer membrane | X |
| *mccB* | Q72V59 | 3-methylcrotonoyl-CoA carboxylase beta subunit | EMJH | Cytoplasm | I |
| *rpiB* | Q72VI0 | Ribose 5-phosphate isomerase B | EMJH | Cytoplasm | G |
| *rlpA* | Q72W83 | Probable endolytic peptidoglycan transglycosylase | EMJH | Outer membrane | M |
| *hemN* | Q75G00 | Coproporphyrinogen-III oxidase | EMJH | Cytoplasm | H |
| *lic20031* | Q75G14 | Short-chain dehydrogenase | EMJH | Outer membrane | S |
| *lic20022* | Q75G27 | Azoreductase | EMJH | Cytoplasm | S |
