# Supplementary material for: Proteomic profile of naturally released extracellular vesicles secreted from Leptospira interrogans serovar Pomona in response to temperature and osmotic stresses
Source: Sci Rep. 2023 Oct 30;13:18601. doi: 10.1038/s41598-023-45863-0 (PMC10616267; doi:10.1038/s41598-023-45863-0)
Supplement: Supplementary file 8 — Supplementary Table S3. [file 41598_2023_45863_MOESM8_ESM.docx]

**S3.1 Table.** The list of 30 most abundant proteins in leptospiral EVs released from leptospires after 30 °C culture in EMJH medium.

| **Abundance ranking** | **Gene Names** | **Protein IDs** | **Protein names** | **Subcellular localization** | **COG** |
| --- | --- | --- | --- | --- | --- |
| 1 | *lic11617* | Q72RX1 | Transcriptional regulator (ArsR family) | Cytoplasm | K |
| 2 | *lic12631* | Q72P45 | Hemolysin | Extracellular | S |
| 3 | *maoC* | Q72SM0 | MaoC | Cytoplasm | I |
| 4 | *groEL* | P61438 | Chaperonin GroEL | Cytoplasm | O |
| 5 | *lipL32* | Q72SM7 | LipL32 | Outer membrane | S |
| 6 | *rpsZ* | Q72NH4 | 30S ribosomal protein S14 type Z | Cytoplasm | J |
| 7 | *lic11958* | Q72QZ4 | ARM repeat superfamily protein | Cytoplasm | X |
| 8 | *lic11687* | Q72RQ7 | Endonuclease | Unknown | S |
| 9 | *lic10411* | Q72V90 | HEAT repeat protein | Unknown | X |
| 10 | *bfr* | Q72SR5 | Bacterioferritin | Cytoplasm | P |
| 11 | *loa22* | Q72VV5 | Peptidoglycan associated Cytoplasm membrane protein | Outer membrane | M |
| 12 | *rpsG* | Q72UA5 | 30S ribosomal protein S7 | Cytoplasm | J |
| 13 | *lic12233* | Q72Q79 | Fructose-bisphosphate aldolase | Cytoplasm | G |
| 14 | *lic12958* | Q72N79 | Transposase | Cytoplasm | X |
| 15 | *lipL21* | Q72WC6 | LipL21 | Outer membrane | X |
| 16 | *lic10970* | Q72TP7 | Acyl-CoA dehydrogenase | Cytoplasm | C |
| 17 | *mucD* | Q72NM1 | Serine protease MucD | Periplasm | O |
| 18 | *lic10235* | Q72VR1 | Profilin domain protein | Cytoplasm | X |
| 19 | *metK* | Q72SM5 | S-adenosylmethionine synthase (AdoMet synthase) | Cytoplasm | H |
| 20 | *rpoA* | Q72NI8 | DNA-directed RNA polymerase subunit alpha (RNAP subunit alpha) | Cytoplasm | K |
| 21 | *pntA* | Q72W91 | Proton-translocating transhydrogenase, subunit alpha part 1 | Cytoplasm | C |
| 22 | *lic11442* | Q72SE1 | Four-helix bundle copper-binding protein | Unknown | X |
| 23 | *lic11209* | Q72T13 | 4HBT domain-containing protein | Cytoplasm | X |
| 24 | *lpxA* | Q72MN9 | Acyl-[acyl carrier protein]--UDP-N-acetylglucosamine O-acyltransferase | Cytoplasm | I |
| 25 | *lipL41* | Q72N71 | LipL41 | Outer membrane | X |
| 26 | *lipL71* | Q72TL5 | LipL71 | Outer membrane | X |
| 27 | *lic10314* | Q72VI3 | P83/100 | Outer membrane | S |
| 28 | *rpoC* | Q72UA7 | DNA-directed RNA polymerase subunit beta' (RNAP subunit beta') | Cytoplasm | K |
| 29 | *rplI* | Q72QK3 | 50S ribosomal protein L9 | Cytoplasm | J |
| 30 | *rpsQ* | Q72NH0 | 30S ribosomal protein S17 | Cytoplasm | J |

**S3.2 Table.** The list of 30 most abundant proteins in leptospiral EVs released from leptospires after 37 °C culture in EMJH medium.

| **Abundance ranking** | **Gene Names** | **Protein IDs** | **Protein names** | **Subcellular localization** | **COG** |
| --- | --- | --- | --- | --- | --- |
| 1 | *maoC* | Q72SM0 | MaoC | Cytoplasm | I |
| 2 | *lic11617* | Q72RX1 | Transcriptional regulator (ArsR family) | Cytoplasm | K |
| 3 | *lic10411* | Q72V90 | HEAT repeat protein | Unknown | X |
| 4 | *lic11687* | Q72RQ7 | Endonuclease | Unknown | S |
| 5 | *groEL* | P61438 | Chaperonin GroEL | Cytoplasm | O |
| 6 | *rpsZ* | Q72NH4 | 30S ribosomal protein S14 type Z | Cytoplasm | J |
| 7 | *lic12631* | Q72P45 | Hemolysin | Extracellular | S |
| 8 | *lic11209* | Q72T13 | 4HBT domain-containing protein | Cytoplasm | X |
| 9 | *lic11958* | Q72QZ4 | ARM repeat superfamily protein | Cytoplasm | X |
| 10 | *lic12233* | Q72Q79 | Fructose-bisphosphate aldolase | Cytoplasm | G |
| 11 | *lic10235* | Q72VR1 | Profilin domain protein | Cytoplasm | X |
| 12 | *rpsG* | Q72UA5 | 30S ribosomal protein S7 | Cytoplasm | J |
| 13 | *pntA* | Q72W91 | Proton-translocating transhydrogenase, subunit alpha part 1 | Cytoplasm | C |
| 14 | *lipL32* | Q72SM7 | LipL32 | Outer membrane | S |
| 15 | *lic12958* | Q72N79 | Transposase | Cytoplasm | X |
| 16 | *rpsQ* | Q72NH0 | 30S ribosomal protein S17 | Cytoplasm | J |
| 17 | *lic10970* | Q72TP7 | Acyl-CoA dehydrogenase | Cytoplasm | C |
| 18 | *lipL41* | Q72N71 | LipL41 | Outer membrane | X |
| 19 | *mucD* | Q72NM1 | Serine protease MucD | Periplasm | O |
| 20 | *lic10314* | Q72VI3 | P83/100 | Outer membrane | S |
| 21 | *metK* | Q72SM5 | S-adenosylmethionine synthase (AdoMet synthase) | Cytoplasm | H |
| 22 | *lipL71* | Q72TL5 | LipL71 | Outer membrane | X |
| 23 | *infC* | Q72PK8 | Translation initiation factor IF-3 | Cytoplasm | J |
| 24 | *bfr* | Q72SR5 | Bacterioferritin | Cytoplasm | P |
| 25 | *rpoA* | Q72NI8 | DNA-directed RNA polymerase subunit alpha (RNAP subunit alpha) | Cytoplasm | K |
| 26 | *rpoC* | Q72UA7 | DNA-directed RNA polymerase subunit beta' (RNAP subunit beta') | Cytoplasm | K |
| 27 | *lic13071* | Q72MW8 | YbhB/YbcL family Raf kinase inhibitor-like protein | Periplasm | S |
| 28 | *katE* | Q72QS7 | Catalase | Periplasm | C |
| 29 | *rpsA* | Q72PM2 | 30S ribosomal protein S1 | Cytoplasm | J |
| 30 | *lic11442* | Q72SE1 | Four-helix bundle copper-binding protein | Unknown | X |

**S3.3 Table.** The list of 30 most abundant proteins in leptospiral EVs released from leptospires after 30 °C culture in EMJH medium supplemented with 120 mM NaCl.

| **Abundance ranking** | **Gene Names** | **Protein IDs** | **Protein names** | **Subcellular localization** | **COG** |
| --- | --- | --- | --- | --- | --- |
| 1 | *lic11617* | Q72RX1 | Transcriptional regulator (ArsR family) | Cytoplasm | K |
| 2 | *groEL* | P61438 | Chaperonin GroEL | Cytoplasm | O |
| 3 | *lic10411* | Q72V90 | HEAT repeat protein | Unknown | X |
| 4 | *lic11958* | Q72QZ4 | ARM repeat superfamily protein | Cytoplasm | X |
| 5 | *lipL32* | Q72SM7 | LipL32 | Outer membrane | S |
| 6 | *maoC* | Q72SM0 | MaoC | Cytoplasm | I |
| 7 | *rpsZ* | Q72NH4 | 30S ribosomal protein S14 type Z | Cytoplasm | J |
| 8 | *lic20035* | Q75G29 | MORN repeat protein | Outer membrane | X |
| 9 | *bfr* | Q72SR5 | Bacterioferritin | Cytoplasm | P |
| 10 | *lic12631* | Q72P45 | Hemolysin | Extracellular | S |
| 11 | *rpoA* | Q72NI8 | DNA-directed RNA polymerase subunit alpha (RNAP subunit alpha) | Cytoplasm | K |
| 12 | *lic11209* | Q72T13 | 4HBT domain-containing protein | Cytoplasm | X |
| 13 | *rpsG* | Q72UA5 | 30S ribosomal protein S7 | Cytoplasm | J |
| 14 | *lipL71* | Q72TL5 | LipL71 | Outer membrane | X |
| 15 | *lipL41* | Q72N71 | LipL41 | Outer membrane | X |
| 16 | *metK* | Q72SM5 | S-adenosylmethionine synthase (AdoMet synthase) | Cytoplasm | H |
| 17 | *pntA* | Q72W91 | Proton-translocating transhydrogenase, subunit alpha part 1 | Cytoplasm | C |
| 18 | *lic12958* | Q72N79 | Transposase | Cytoplasm | X |
| 19 | *rpoC* | Q72UA7 | DNA-directed RNA polymerase subunit beta' (RNAP subunit beta') | Cytoplasm | K |
| 20 | *lpxA* | Q72MN9 | Acyl-[acyl carrier protein]--UDP-N-acetylglucosamine O-acyltransferase | Cytoplasm | I |
| 21 | *lic10235* | Q72VR1 | Profilin domain protein | Cytoplasm | X |
| 22 | *lic10175* | Q72VX0 | Tail sheath protein | Cytoplasm | X |
| 23 | *argS* | Q72QL1 | Arginine--tRNA ligase | Cytoplasm | J |
| 24 | *lic10970* | Q72TP7 | Acyl-CoA dehydrogenase | Cytoplasm | C |
| 25 | *fadB* | Q72M90 | 3-hydroxyacyl-CoA dehydrogenase | Cytoplasm | I |
| 26 | *loa22* | Q72VV5 | Peptidoglycan associated Cytoplasm membrane protein | Outer membrane | M |
| 27 | *infC* | Q72PK8 | Translation initiation factor IF-3 | Cytoplasm | J |
| 28 | *lipL21* | Q72WC6 | LipL21 | Outer membrane | X |
| 29 | *rpmC* | Q72NG9 | 50S ribosomal protein L29 | Cytoplasm | X |
| 30 | *lic11687* | Q72RQ7 | Endonuclease | Unknown | S |
