# Supplementary material for: Proteomic profile of naturally released extracellular vesicles secreted from Leptospira interrogans serovar Pomona in response to temperature and osmotic stresses
Source: Sci Rep. 2023 Oct 30;13:18601. doi: 10.1038/s41598-023-45863-0 (PMC10616267; doi:10.1038/s41598-023-45863-0)
Supplement: Supplementary file 9 — Supplementary Table S4. [file 41598_2023_45863_MOESM9_ESM.docx]

**S4 Table.** The list of proteins with relative abundance changes in response to temperature shift.

| **Gene Names** | **Protein IDs** | **Protein names** | **Average log2 ratio** | **p value** | **Subcellular localization** | **COG** |
| --- | --- | --- | --- | --- | --- | --- |
| *tktA* | Q72TV3 | Transketolase alpha subunit protein | 2.967 | 0.003 | Cytoplasm | G |
| *pykF* | Q75FD0 | Pyruvate kinase | 2.567 | 0.019 | Cytoplasm | G |
| *lic10808* | Q72U56 | 4HBT domain-containing protein | 2.523 | 0.003 | Cytoplasm | Q |
| *lic20100* | Q75FT1 | HDOD domain-containing protein | 2.406 | 0.020 | Cytoplasm | X |
| *lic10984* | Q72TN3 | FGE-sulfatase domain-containing protein | 2.322 | 0.036 | Cytoplasm | X |
| *lic10138* | Q72W03 | HD-GYP hydrolase domain protein | 2.206 | 0.007 | Cytoplasm | X |
| *rpsT* | Q72V55 | 30S ribosomal protein S20 | 2.185 | 0.005 | Cytoplasm | J |
| *lic13022* | Q72N16 | PF07075 family protein | 2.124 | 0.027 | Cytoplasm | S |
| *lic12341* | Q72PX6 | Toluene tolerance protein | 1.935 | 0.004 | Outer membrane | X |
| *lic13240* | Q72ME8 | Nucleoside triphosphate pyrophosphohydrolase | 1.872 | 0.036 | Cytoplasm | S |
| *lic12008* | Q72QV0 | Sugar O-acyltransferase, sialic acid O-acetyltransferase NeuD family | 1.853 | 0.015 | Cytoplasm | X |
| *lic12303* | Q72Q11 | Carboxy-terminal processing protease | 1.844 | 0.006 | Unknown | M |
| *lic20001* | Q75G12 | F5/8 type C domain-containing protein | 1.837 | 0.001 | Outer membrane | X |
| *lic20265* | Q75FC9 | EF-hand domain-containing protein | 1.766 | 0.013 | Cytoplasm | X |
| *murA* | Q72MD7 | UDP-N-acetylglucosamine 1-carboxyvinyltransferase | 1.689 | 0.023 | Cytoplasm | M |
| *gatA* | Q72SC3 | Glutamyl-tRNA(Gln) amidotransferase subunit A | 1.556 | 0.044 | Cytoplasm | J |
| *lic13206* | Q72MI1 | 4HBT domain-containing protein | 1.536 | 0.005 | Unknown | Q |
| *gshA* | Q72RD4 | Glutamate--cysteine ligase | 1.425 | 0.009 | Cytoplasm | H |
| *lic12421* | Q72PP7 | ATPase, AAA family | 1.423 | 0.045 | Cytoplasm | X |
| *rimP* | Q72NX1 | Ribosome maturation factor RimP | 1.414 | 0.006 | Cytoplasm | S |
| *lic12073* | Q72QP0 | DUF4065 domain-containing protein | 1.380 | 0.024 | Cytoplasm | X |
| *lic12796* | Q72NN6 | Thioesterase-like family protein | 1.325 | 0.029 | Cytoplasm | X |
| *lic20067* | Q75FW4 | MutT-like protein | 1.323 | 0.019 | Cytoplasm | X |
| *purQ* | Q72UH6 | Phosphoribosylformylglycinamidine synthase subunit PurQ | 1.268 | 0.038 | Cytoplasm | F |
| *oppA* | Q72TK6 | OppA | 1.217 | 0.019 | Unknown | E |
| *mtnB* | Q75FG3 | Methylthioribulose-1-phosphate dehydratase | 1.191 | 0.005 | Cytoplasm | E |
| *lic11534* | Q72S52 | Corrinoid adenosyltransferase | 1.145 | 0.001 | Cytoplasm | S |
| *lic10444* | Q72V58 | Glucose-1-phosphate thymidylyltransferase | 0.944 | 0.033 | Cytoplasm | M |
| *purH* | Q72RT5 | Bifunctional purine biosynthesis protein PurH | -0.841 | 0.015 | Cytoplasm | F |
| *lic20197* | Q75FI7 | Cysteine protease | -0.975 | 0.021 | Outer membrane | U |
| *lic10927* | Q72TT9 | Putative lipoprotein | -1.088 | 0.041 | Extracellular | X |
| *glnB* | Q72V62 | Nitrogen regulatory protein pII | -1.431 | 0.027 | Cytoplasm | K |
| *lic11463* | Q72SC1 | PGAP1-like protein | -1.836 | 0.024 | Unknown | X |
| *ligA* | G1UB65 | Ig-like repeat domain protein 1 | -1.861 | 0.021 | Outer membrane | N |
| *ligB* | Q72V39 | Ig-like repeat domain protein 3 | -1.999 | 0.013 | Outer membrane | N |
| *flaB* | Q72R58 | Flagellin | -2.006 | 0.001 | Cytoplasm | N |
| *ompL47* | Q72MY9 | Bacterial group 3 Ig-like protein | -2.013 | 0.007 | Outer membrane | X |
| *lic11499* | Q72S85 | DUF1931 domain-containing protein | -2.415 | 0.017 | Cytoplasm | X |
| *rplL* | Q72UA9 | 50S ribosomal protein L7/L12 | -3.584 | 0.025 | Unknown | J |
