# Supplementary material for: Proteomic profile of naturally released extracellular vesicles secreted from Leptospira interrogans serovar Pomona in response to temperature and osmotic stresses
Source: Sci Rep. 2023 Oct 30;13:18601. doi: 10.1038/s41598-023-45863-0 (PMC10616267; doi:10.1038/s41598-023-45863-0)
Supplement: Supplementary file 10 — Supplementary Table S5. [file 41598_2023_45863_MOESM10_ESM.docx]

**S5 Table.** The list of proteins with relative abundance changes in response to physiologic osmolarity.

| **Gene Names** | **Protein IDs** | **Protein names** | **Average log2 ratio** | **p value** | **Subcellular localization** | **COG** |
| --- | --- | --- | --- | --- | --- | --- |
| *lic13257* | Q72MD1 | NADH oxidoreductase | 1.605 | 0.005 | Cytoplasm | C |
| *tkta* | Q72TV3 | Transketolase alpha subunit protein | 1.521 | 0.030 | Cytoplasm | G |
| *lic1080* | Q72U56 | 4HBT domain-containing protein | 1.096 | 0.011 | Cytoplasm | Q |
| *lic13240* | Q72ME8 | Nucleoside triphosphate pyrophosphohydrolase | 0.900 | 0.013 | Cytoplasm | S |
| *manB* | Q72RH9 | Phosphomannomutase | 0.696 | 0.004 | Cytoplasm | G |
| *lic10799* | Q72U65 | Enoyl-CoA hydratase | -0.314 | 0.000 | Unknown | X |
| *lic11958* | Q72QZ4 | ARM repeat superfamily protein | -0.365 | 0.025 | Cytoplasm | X |
| *lic12561* | Q72PB1 | Secreted protein | -0.455 | 0.008 | Unknown | X |
| *lic12339* | Q72PX8 | PF07598 family protein | -0.492 | 0.041 | Inner membrane | S |
| *lic10138* | Q72W03 | HD-GYP hydrolase domain protein | -0.504 | 0.033 | Cytoplasm | X |
| *ivd* | Q72VD5 | Isovaleryl-CoA dehydrogenase | -0.572 | 0.025 | Cytoplasm | C |
| *bfr* | Q72SR5 | Bacterioferritin | -0.615 | 0.028 | Cytoplasm | P |
| *rnD* | Q72MC5 | Ribonuclease D | -0.777 | 0.027 | Cytoplasm | J |
| *lolA* | Q72PC6 | Outer membrane lipoprotein carrier protein | -0.778 | 0.030 | Unknown | X |
| *lruC* | Q75FL0 | Lipoprotein | -0.805 | 0.014 | Outer membrane | X |
| *lic10970* | Q72TP7 | Acyl-CoA dehydrogenase | -0.867 | 0.036 | Cytoplasm | C |
| *sch1* | Q72PZ8 | Beta-ketoacyl synthase | -0.884 | 0.007 | Cytoplasm | I |
| *rpsJ* | Q72NG0 | 30S ribosomal protein S10 | -0.891 | 0.039 | Cytoplasm | J |
| *hbd1* | Q72UU2 | Enoyl-CoA hydratase | -0.934 | 0.005 | Cytoplasm | X |
| *ileS* | Q72PR7 | Isoleucine--tRNA ligase | -0.940 | 0.014 | Cytoplasm | J |
| *lic11888* | Q72R60 | Glycoside hydrolase family 8 | -0.979 | 0.009 | Unknown | X |
| *lic10764* | Q72U97 | UPF0234 protein LIC_10764 | -1.004 | 0.024 | Cytoplasm | S |
| *glnA* | Q72PR0 | Putative glutamine synthetase protein | -1.006 | 0.009 | Cytoplasm | E |
| *capA* | Q72PU0 | Capsule biosynthesis protein | -1.012 | 0.015 | Unknown | M |
| *lic12324* | Q72PZ1 | Non-specific serine/threonine protein kinase | -1.029 | 0.016 | Outer membrane | X |
| *lic12730* | Q72NU9 | NHL repeat protein | -1.037 | 0.008 | Outer membrane | X |
| *dps* | Q72UQ1 | DNA-binding stress protein | -1.048 | 0.033 | Cytoplasm | P |
| *lic13071* | Q72MW8 | YbhB/YbcL family Raf kinase inhibitor-like protein | -1.062 | 0.034 | Periplasm | S |
| *lic11141* | Q72T80 | Ribonuclease D | -1.079 | 0.000 | Cytoplasm | L |
| *aspC* | Q72RF5 | Aminotransferase | -1.104 | 0.024 | Cytoplasm | E |
| *groEL* | P61438 | Chaperonin GroEL | -1.109 | 0.007 | Cytoplasm | O |
| *btuE* | Q72P28 | Glutathione peroxidase | -1.147 | 0.007 | Periplasm | O |
| *tlyB* | Q72VF8 | Hemolysin B | -1.150 | 0.008 | Cytoplasm | O |
| *lic10768* | Q72U93 | Metalloprotease | -1.169 | 0.017 | Cytoplasm | S |
| *rpsA* | Q72PM2 | 30S ribosomal protein S1 | -1.203 | 0.027 | Cytoplasm | J |
| *clpB* | Q72QU2 | Chaperone protein ClpB | -1.205 | 0.018 | Cytoplasm | O |
| *lic10760* | Q72UA1 | YceI domain-containing protein | -1.209 | 0.009 | Unknown | S |
| *lic11273* | Q72SV0 | PF09986 family protein | -1.300 | 0.000 | Cytoplasm | S |
| *lic20185* | Q75FJ7 | FecR domain-containing protein | -1.305 | 0.024 | Outer membrane | X |
| *lic10769* | Q72U92 | Processing metalloprotease | -1.342 | 0.015 | Unknown | X |
| *lic12645* | Q72P31 | A2M domain-containing protein | -1.377 | 0.017 | Outer membrane | X |
| *lic11518* | Q72S68 | Biotin carboxyl carrier protein of acetyl-CoA carboxylase | -1.387 | 0.001 | Cytoplasm | I |
| *lic11672* | Q72RS0 | Enoyl-CoA hydratase | -1.429 | 0.004 | Cytoplasm | I |
| *sseA* | Q72TA6 | Thiosulfate sulfurtransferase | -1.439 | 0.025 | Cytoplasm | X |
| *lic12250* | Q72Q63 | Carboxy-terminal processing protease | -1.479 | 0.007 | Unknown | M |
| *accA2* | Q72S69 | Acetyl-CoA carboxylase alpha subunit | -1.482 | 0.004 | Cytoplasm | I |
| *purH* | Q72RT5 | Bifunctional purine biosynthesis protein PurH | -1.599 | 0.038 | Cytoplasm | F |
| *lic12318* | Q72PZ7 | ATP-binding protein | -1.612 | 0.008 | Cytoplasm | X |
| *rplO* | Q72NI0 | 50S ribosomal protein L15 | -1.650 | 0.043 | Cytoplasm | J |
| *atpA* | Q72SY1 | ATP synthase subunit alpha | -1.662 | 0.004 | Cytoplasm | F |
| *pnp* | Q72NX7 | Polyribonucleotide nucleotidyltransferase | -1.690 | 0.003 | Cytoplasm | J |
| *ahpC* | Q72T03 | Peroxiredoxin | -1.723 | 0.000 | Cytoplasm | O |
| *lic11194* | Q72T27 | Putative citrate lyase | -1.774 | 0.000 | Cytoplasm | G |
| *mucD* | Q72NM1 | Serine protease MucD | -1.792 | 0.000 | Periplasm | O |
| *lpdA* | Q72PJ6 | Dihydrolipoyl dehydrogenase | -1.796 | 0.026 | Cytoplasm | C |
| *mgsA* | Q72NU6 | Methylglyoxal synthase (MGS) | -1.923 | 0.026 | Cytoplasm | G |
| *lic12233* | Q72Q79 | Fructose-bisphosphate aldolase | -2.031 | 0.001 | Cytoplasm | G |
| *lic13128* | Q72MR5 | TerB family tellurite resistance protein | -2.046 | 0.003 | Cytoplasm | X |
| *lic11728* | Q72RL7 | N-acetylmuramoyl-L-alanine amidase | -2.129 | 0.000 | Cytoplasm | M |
| *lic11687* | Q72RQ7 | Endonuclease | -2.150 | 0.000 | Unknown | S |
| *ompL36* | Q72MM7 | SH3 domain-containing protein | -2.268 | 0.003 | Outer membrane | X |
| *phoD* | Q72LZ6 | Phosphodiesterase | -2.274 | 0.001 | Unknown | P |
| *lic11423* | Q72SG0 | Glycine rich RNA-binding protein | -2.424 | 0.007 | Unknown | S |
| *flaB* | Q72R58 | Flagellin | -2.532 | 0.019 | Cytoplasm | N |
| *ligB* | Q72V39 | Ig-like repeat domain protein 3 | -2.574 | 0.001 | Outer membrane | N |
| *lic12631* | Q72P45 | Hemolysin | -2.596 | 0.002 | Extracellular | S |
| *lic10829* | Q72U35 | Leucine rich repeat protein | -2.788 | 0.019 | Extracellular | X |
| *ligA* | G1UB65 | Ig-like repeat domain protein 1 | -2.936 | 0.005 | Outer membrane | N |
| *glnB* | Q72V62 | Nitrogen regulatory protein pII | -3.856 | 0.000 | Cytoplasm | K |
